# The *Nasonia* pair-rule gene regulatory network retains its function over 300 million years of evolution

**DOI:** 10.1242/dev.199632

**Published:** 2022-03-09

**Authors:** Shannon E. Taylor, Peter K. Dearden

**Affiliations:** Genomics Aotearoa and Department of Biochemistry, University of Otago, PO Box 56, Dunedin 9016, Aotearoa-New Zealand

**Keywords:** Gene regulatory network, Pair-rule genes, Patterning, Segmentation, *Nasonia*

## Abstract

Insect segmentation is a well-studied and tractable system with which to investigate the genetic regulation of development. Though insects segment their germband using a variety of methods, modelling work implies that a single gene regulatory network can underpin the two main types of insect segmentation. This means limited genetic changes are required to explain significant differences in segmentation mode between different insects. This idea needs to be tested in a wider variety of species, and the nature of the gene regulatory network (GRN) underlying this model has not been tested. Some insects, e.g. *Nasonia vitripennis* and *Apis mellifera* segment progressively, a pattern not examined in previous studies of this segmentation model, producing stripes at different times progressively through the embryo, but not from a segment addition zone. Here, we aim to understand the GRNs patterning *Nasonia* using a simulation-based approach. We found that an existing model of *Drosophila* segmentation ( Clark, 2017) can be used to recapitulate the progressive segmentation of *Nasonia*, if provided with altered inputs in the form of expression of the timer genes *Nv-caudal* and *Nv-odd paired*. We predict limited topological changes to the pair-rule network and show, by RNAi knockdown, that *Nv-odd paired* is required for morphological segmentation. Together this implies that very limited changes to the *Drosophila* network are required to simulate *Nasonia* segmentation, despite significant differences in segmentation modes, implying that *Nasonia* use a very similar version of an ancestral GRN used by *Drosophila*, which must therefore have been conserved for at least 300 million years.

## Abstract (precis)

**Summary:** The gene regulatory network that controls segmentation in the wasp *Nasonia* is functionally similar to that of *Drosophila*, despite different modes of segmentation and 300 million years of divergence.

## INTRODUCTION

The genetic and developmental changes required for phenotypic evolution are an enduring question in evo-devo. Arthropod segmentation is a classic system in which to study this question, in large part because of the enormous variation in methods of segmentation (see Akam, 1987; Davis and Patel, 2002; Peel and Akam, 2003; Clark et al., 2019; Chipman, 2020 for reviews). Insect segmentation is best understood in *Drosophila melanogaster*, where two protein gradients, of bicoid (bcd) and caudal (cad), along with other maternally provided proteins, establish the early anterior-posterior pattern. Proteins encoded by gap genes then subdivide the body axis into broad domains, and regulate expression of the pair-rule genes (PRGs), marking the first periodic gene expression in the embryo. So-called primary PRGs are regulated by gap genes and each other, whereas secondary PRGs are regulated only by the primary PRGs (Schroeder et al., 2011; Clark et al., 2019). Both primary and secondary pair-rule genes regulate the segment polarity genes to produce the periodic, segmental output of the pair-rule system (Schroeder et al., 2011; Clark et al., 2019). These segment polarity genes position the boundaries of morphological segmentation.

*Drosophila* produce each body segment at the same time within the embryo, hence segmenting simultaneously. Many other insects segment sequentially, producing new segments one after the other, from a posterior segment addition zone (Davis and Patel, 2002; Peel and Akam, 2003; Clark et al., 2019). These two modes of segmentation exist on a spectrum (Peel and Akam, 2003; Clark et al., 2019). For example, *Nasonia vitripennis*, the subject of this study, and the honeybee *Apis mellifera* express segment polarity and pair-rule genes in an anterior to posterior progression (Fleig and Sander, 1988; Binner and Klaus, 1997; Osborne and Peter, 2005; Wilson and Peter, 2012; Rosenberg et al., 2014). That is, stripes of segmentation gene expression appear *de novo*, one after the other, but are not produced from the posterior of the embryo. Progressive segmentation, is then an intermediate between simultaneous and sequential segmentation. This progressive segmentation probably evolved independently from that of *Drosophila*, as *Nasonia* and the honeybee are basal to *Drosophila* (simultaneous) and *Tribolium* (sequential).

Recent work has shed light on how sequential and simultaneous segmentation could evolve and co-exist (as in *Nasonia*). The *Drosophila* pair-rule GRN is best understood as two networks: early and late (Clark and Michael, 2016). The early network produces periodic, pair-rule gene expression, while the late network converts this pair-rule pattern into the segment polarity pattern (Clark, 2017). This relies on forward shifts in pair-rule gene expression, which could be driven by forward movement of gap genes or by an oscillatory pair-rule network.

Changing the timing of network activation may control whether segmentation is simultaneous or sequential. If the timing of network activation is the same across the whole embryo, simultaneous segmentation occurs as each segment matures at the same time. If the networks are activated at different times throughout the embryo, the embryo segments sequentially (Clark, 2017; Clark and Andrew, 2018). The initial pattern could come from periodic pair-rule gene expression (a segmentation clock) and/or gap gene expression.

Transitions between these networks are thought to be controlled by the timer genes *cad*, *Dichaete* (*D*), *Sox21b* and *odd-paired* (*opa*). *cad* has a well-conserved role as a posterior determinant, and regulates pair-rule gene expression in many species (Copf et al., 2004; Wilson et al., 2010; Kimelman and Benjamin, 2012; El-Sherif et al., 2014; Rosenberg et al., 2014; Schönauer et al., 2016; Zhu et al., 2017; Novikova et al., 2020). *D* (or *Sox21b* in spiders) is required for normal expression of some pair-rule genes in *Drosophila* and for spider segmentation, and the expression of *D* orthologues in the segment addition zone is conserved throughout pan-arthropoda (Nambu and Nambu, 1996; Russell et al., 1996; Ma et al., 1998; Clark and Andrew, 2018; Janssen et al., 2018; Paese et al., 2018; Baudouin-Gonzalez et al., 2020 preprint). In *Drosophila*, *opa* is required for late network activation and genome-wide regulatory changes (Clark and Michael, 2016; Koromila et al., 2020; Soluri et al., 2020). In other insects, *opa* is expressed in a band at the end of the segment addition zone, where a putative late network would be active, although *Oncopeltus* lack this pattern, implying this timer gene role may not be conserved across all insects (Green and Michael, 2013; Xiang et al., 2017; Janssen et al., 2011; Auman and Ariel, 2018).

The timer gene proposal has important implications. First, it provides a simple way to evolve phenotypic diversity: expression patterns of timer genes controls the difference between sequential and simultaneous segmentation, and implies that relatively few regulatory changes can have dramatic phenotypic consequences (Clark, 2017; Clark and Andrew, 2018; Clark et al., 2019). Second, it provides a biological example of a multifunctional GRN. The term ‘function’ has multiple meanings in biology. It can refer to what a trait, organ or GRN possesses [e.g. the beating of the heart or the oscillatory versus stable behaviour of a GRN (activity-function; Wouters, 2003)]. Alternatively, it can refer to how a trait is used by the organism, e.g. the heart moving blood around the body or a GRN patterning segments of an insect (use-function; Wouters, 2003). ‘Function’ can also refer to what a trait was selected for or, alternatively, how it confers an advantage to a given organism (Wouters, 2003). The multifunctionality of GRNs modelled as ordinary differential equations is well documented. Most dramatically, the AC/DC circuit (three genes in a circuit, each repressing the one after, and one pair of genes also repressing each other) is capable of both oscillations and multistable behaviour [i.e. it has multiple activity-functions (Panovska-Griffiths et al., 2013; Perez-Carrasco et al., 2018; Verd et al., 2019)]. Gene circuits with the same topology are capable of different dynamic behaviours when the weights of interactions between genes are changed (Jiménez et al., 2017). These are examples of differences in activity-function. These different behaviours are central to explaining the evolvability of the gap gene system of flies: they have different functions (Verd et al., 2019). The pair-rule system provides another example of a multifunctional GRN. Both sequential and simultaneous segmentation rely on the same activity-function: periodic PRG expression (driven by gap or PRGs). However, how this is used to produce sequential versus simultaneous patterning – the use-function – has changed.

We wished to understand how the activity-function of the pair-rule network may or may not have changed to achieve the different use-function (progressive segmentation) in *Nasonia*. *Nasonia vitripennis* is a parasitoid wasp: a hymenopteran insect that diverged from *Drosophila* ∼300 million years ago. The evolutionary distance of *Nasonia* from *Drosophila*, the differences in life history between these species and the morphological differences in segmentation between them provide an opportunity to understand how GRNs evolve to produce different developmental patterns. We are interested in: (1) how progressive patterning could be achieved in *Nasonia*; (2) whether the *Nasonia* pair-rule GRN is organised like that of *Drosophila*; and (3) how well the *Drosophila* network topology can recapitulate changes in *Nasonia* pair-rule gene expression. We approach this by producing a precisely staged description of *Nasonia* segmentation, then adapting Clark's model of *Drosophila* pair-rule patterning to recapitulate these patterns (Clark, 2017). This computational model idealises the embryo as a one-dimensional row of cells, obeying Boolean logic to determine how gene expression (protein and RNA expression and age) changes over time (Clark, 2017). Overall, despite the biological differences in patterning of *Nasonia*, we find that surprisingly few changes to the *Drosophila* pair-rule GRN are required to simulate *Nasonia*-like patterning, implying that there may be limited changes to this network throughout insect evolution.

## RESULTS

### Dynamics of segmentation gene expression in *Nasonia*

**Fig. 1.**
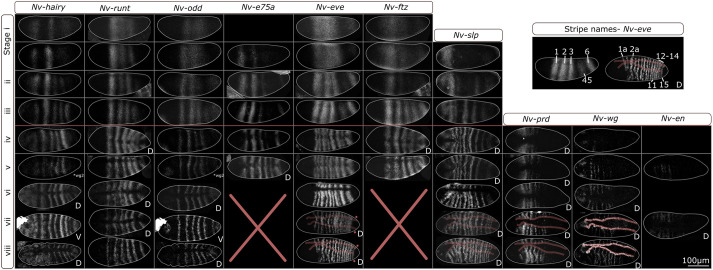
**Pair-rule gene expression during *Nasonia* development.** All embryos are maximum intensity projections (half or full embryos), oriented anterior left and dorsal up. Embryos are staged by the number of *Nv-eve* or *wg* stripes. Some embryos are re-used, e.g. *odd* and *hairy* expression comes from the same embryo. Embryos are laterally oriented unless otherwise indicated (D, dorsal orientation; V, ventral orientation). The boundary of the ectoderm (demarcated by *Nv-sim*) is highlighted in red; red stars indicate *Nv-eve* stripe 15. The top right-hand image indicates stripe names according to Rosenberg et al. (2014).

To compare *Nasonia* and *Drosophila* gene expression during segmentation, we needed a comprehensive and precisely staged description of *Nasonia* segmentation. To enable robust temporal and spatial characterisation of gene expression, we compared expression of each pair-rule gene to the expression of *Nv-eve* and *Nv-wg*, alongside *Nv-sim* (a marker of the ectoderm/mesoderm boundary, Buchta et al., 2013) for selected genes. We stained for the known *Drosophila* pair-rule genes, plus *Nv-e75A*, which is involved in *Oncopeltus* segmentation (Erezyilmaz et al., 2009; Reding et al., 2019). All of these expression patterns, except for *Nv-e75A* and *Nv-slp*, have been previously described; comprehensive descriptions are available for *Nv-eve*, *Nv-odd*, *Nv-hairy*, *Nv-runt*, *Nv-wg* and *Nv-prd* (Rosenberg et al., 2014; Keller et al., 2010; Olesnicky and Claude, 2007). We build upon this work by elaborating the timing of these events, by reporting/elaborating the expression of *Nv-slp* and *Nv-ftz*, and by detailing the relative expressions of genes within the embryo. We staged embryos by the number of *wg*/*eve* stripes, as shown in Fig. 1.

*Nasonia* exhibit a similar sequence of patterning events to those in *Drosophila*. Primary pair-rule genes (*hairy*, *odd*, *runt*, *eve* and *ftz*) are expressed first, followed by *slp*. Although *prd* is a secondary pair-rule gene in *Drosophila*, *Nv-prd* is expressed with the other segment polarity genes *en* and *wg*. We also stained for *Nv-e75a*, which is expressed in a pair-rule pattern. This sequence of gene expression holds in each region of the embryo. The primary pair-rule genes do not resolve at the same time: in all regions of the embryo, *hairy* is the first gene to be segmentally expressed, followed by *Nv-odd*, *Nv-E75A* and *Nv-ftz*, then *Nv-eve*.

**Fig. 2.**
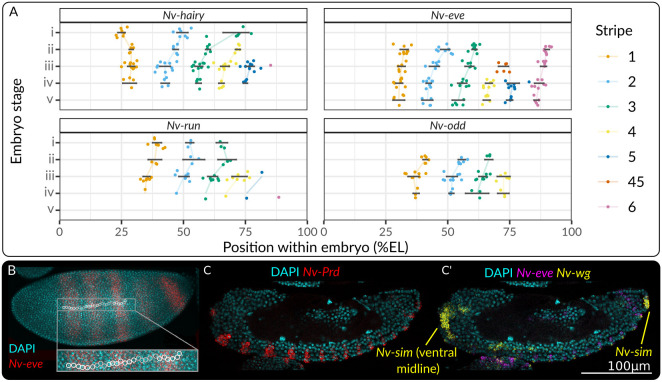
**Dynamics of pair-rule gene expression in *Nasonia*.** (A) Forward movement of pair-rule stripes of gene expression within the embryo over time. Central positions of stripes were gauged by eye from straightened intensity profiles along the middle of the embryo. Black bars indicate ±s.e.m.; coloured lines join the mean at different stages. (B) Maximum intensity projection of a stage iii embryo. White circles surround cell nuclei, demonstrating that each *eve* stripe is six cells wide. The enlargement illustrates this more clearly. (C,C′) *Nasonia* segment polarity genes are expressed in one-cell wide stripes within a four-cell repeat, as in *Drosophila* and other species. Images are single slices through the middle of the embryo.

We also observed that *Nasonia Nv-eve* RNA stripes were five to seven nuclei wide, in contrast to the three or four cell stripes in *Drosophila* (Schroeder et al., 2011). This pattern shrinks: mature *Nv-wg* RNA stripes are one cell wide and separated by three nuclei (Fig. 2C,D; Fig. S2). It is unclear how and why this occurs.

In agreement with Rosenberg et al. (2014), we identified three distinct regions of the *Nasonia* embryo. In the anterior (*eve* stripes 1-5), *Nasonia* undergo progressive segmentation. Pair-rule stripes of *Nv-hairy*, *Nv-odd*, *Nv-E75A* and *Nv-ftz* are expressed in anterior to posterior progression. The expression of *Nv-runt* and *Nv-eve* stripes is simultaneous: the first three stripes appear at around the same time, with *Nv-runt* being expressed first (around the same time as *Nv-odd*). Frequency doubling of stripes of *Nv-slp* and *Nv-eve* RNA expression commences at stage iv and proceeds anterior to posterior, being finished in the 5th eve stripe by stage viii (Rosenberg et al., 2014, Fig. 1). The segment polarity genes *Nv-wg Nv-en* and *Nv-prd* likewise express RNA in stripes that form from anterior to posterior, and are only expressed in the ectoderm (as indicated by *Nv-sim* expression), meaning that only the ectoderm autonomously segments in *Nasonia* (Fig. 1). Together, these data show that anterior *Nasonia* segmentation is progressive, although the expression of *Nv-eve* and *Nv-runt* are exceptions to this.

A region towards the posterior of the embryo, at 85-90% EL, in the region of the sixth *eve* RNA stripe, segments differently to the rest of the embryo. *Nv-e75A* is expressed within this region until at least stage v (we did not image *Nv-e75A* stained embryos later than this), but other genes are not expressed in this stripe. The first segmental expression to be detected in this region of the embryo is that of *Nv-hairy* RNA, at stage vi (Fig. 1). Shortly after this, *Nv-eve* RNA stripe 11 emerges from the anterior of the 6th *eve* RNA stripe (Rosenberg et al., 2014, Fig. 1). Faint segmental expression of *Nv-slp* RNA is then observed, followed by splitting of *eve* RNA stripe six into three stripes of single segment periodicity, separated by a stripe of *Nv-slp* RNA expression (Fig. S3). A sixth *Nv-odd* RNA stripe is detectable at this stage, but expression is delayed and very faint (Fig. S3, Rosenberg et al., 2014).

The posterior region of *Nasonia* embryos segments sequentially. The first expression of pair-rule gene RNA in this region is in a posterior cap of *Nv-odd* RNA, present from stage ii (Rosenberg et al., 2014, Fig. 1). *Nv-hairy* RNA is expressed faintly and inconsistently at stage iv. Stripes of *Nv-ftz* and *Nv-runt* RNA appear posterior to the sixth *eve* stripe. Later, at stage v, the posterior *Nv-hairy* RNA expression becomes stronger and *Nv-odd* RNA is excluded from the *Nv-hairy* expression domain (Fig. S3). After this, the 15th *eve* stripe appears in the posterior, appearing considerably thinner than the other stripes. This is the only sequentially appearing *Nv-eve* RNA stripe we observed, although Rosenberg et al., 2014 observe a 16th (possibly sequential) stripe later. The 16 stripes of the *Nasonia* are then established. During segmentation, key embryological events are taking place: cellularisation and gastrulation. Cellularisation does not commence in *Nasonia* until at least stage iv, meaning early segmentation occurs in a pre-cellular environment (Fig. S1). The embryo begins to gastrulate after the establishment of pair-rule stripes, including the 15th (Fig. 1, Figs S6, S7 and S8). This means that *Nasonia* is a long germband insect, patterning all segments before gastrulation (Davis and Patel, 2002).

### *Nasonia* timer gene expression can produce progressive patterning

We wished to explain the progressive patterning of *Nasonia*, and the compression of the pair rule pattern over time. We first stained for the proposed regulators of different phases of segmentation (*cad*, *D* and *opa*) (Clark and Michael, 2016; Clark, 2017; Clark and Andrew, 2018), and used these patterns as inputs to Clark's model of pair-rule patterning (Clark, 2017).

**Fig. 3.**
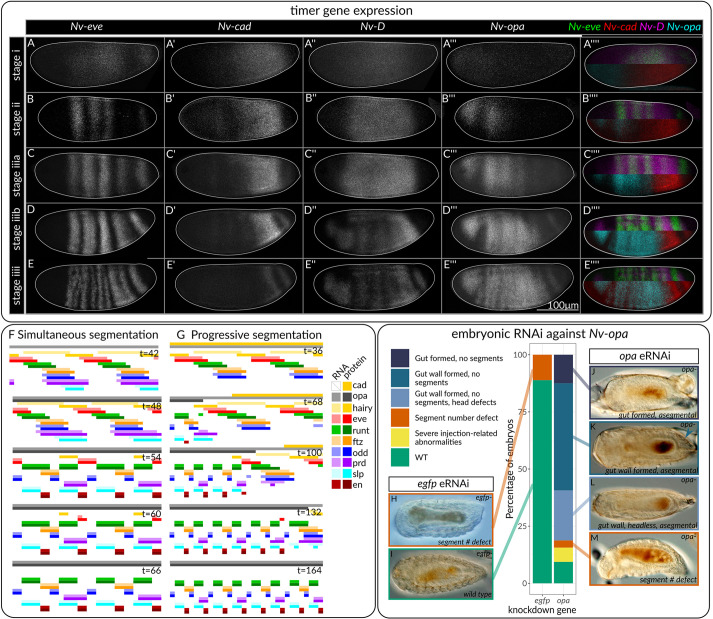
**Timer gene expression in *Nasonia* recapitulates progressive patterning when modelled.** (A-E″″) Maximum intensity projections of embryos stained with the timer genes *Nv-cad*, *Nv-D* and *Nv-opa*, staged to *Nv-eve*. All embryos are laterally oriented with anterior leftwards. In A″″,B″″,C″″,D″″,E″″, *D* and *eve* are shown in the top of the embryo, and *opa* and *cad* in the bottom. (F,G) Simulation of broad pair-rule stripes combined with simultaneous (F) and sequential/progressive (G) timer gene dynamics. Simulations were performed within the framework of Clark (2017), which idealises the embryo as a one-dimensional row of cells, where genes obey Boolean logical rules to update gene expression. (H-M) RNA interference targeting *Nv-opa* demonstrate that it is required for morphological segmentation. RNAi injection produces a range of phenotypes (J-M) that differ from those caused by injection of a control dsRNA (H,I). Sixty-nine percent of *Nv-opa* eRNAi-injected embryos show loss of segmentation. All embryos are oriented anterior leftwards, ventral view.

In the first five *eve* RNA stripes, *Nv-cad* RNA retracts across the anterior-posterior axis as *Nv-opa* RNA expands (Fig. 3A-E, Olesnicky et al., 2006). *Nv-cad* RNA retracts from the anterior (30-65%EL) region before segmental expression of *Nv-eve*, and continues to retract towards the posterior as *eve* stripe45 splits (Fig. 3A,B, Fig. S5). *Nv-opa* RNA is first detected in the head and stripe 1 at stage ii, and expands posteriorly at stage iii, shortly before *Nv-eve* stripe splitting (Fig. 3E). *Nv-D* is expressed differently. At stage ii, it is expressed in a broad band, from the anterior of stripe 1 to the anterior of stripe 6. This expression persists until shortly before *eve* frequency doubling; at this time, *Nv-D* expression is lost in stripes 1-3 in a pair-rule-like pattern strongly resembling *Dm-D* (Nambu and Nambu, 1996; Russell et al., 1996). *Nv-D* expression is retained around stripe4/5 until these stripes start to undergo frequency doubling. Curiously, the middle region of the *Nasonia* embryo, stripe 6, does not follow the above sequence of timer genes, either spatially or temporally. In this region of the embryo, *Nv-cad* and *Nv-D* RNA are never expressed; only *Nv-opa* RNA. Here, as in other regions of the embryo, *Nv-hairy* is the first RNA expression we detected as segmentally expressed, and *Nv-slp* RNA segmental expression slightly precedes *Nv-eve* RNA segmental expression, implying that a similar regulatory hierarchy controls stripe 6 and other regions of the *Nasonia* embryo. However, how this stripe achieves stripe splitting without *cad* and *D* is unclear.

These *opa* and *cad* expression dynamics were able to recapitulate *Nasonia*-like progressive patterning if simulated in a model containing the broad (six-cell wide) pair-rule stripes of *Nasonia* (Fig. 3F,G). In this model, stripes mature in an anterior to posterior progression, characteristic of progressive segmentation. The *cad* and *opa* dynamics are crucial to this progressive patterning: the same network simulated with broad pair-rule stripes, but activated in a simultaneous manner results in an embryo that segments simultaneously, but has a final pattern doubled in size from the *Drosophila* model, i.e. a 16-cell repeat of gene expression (Fig. 3F). The progressive model exhibits the *Drosophila* and *Nasonia*-like eight-cell repeat. The phasing of the late network in this simulation is the same as that in *Drosophila*, but is not *Nasonia*-like.

To further investigate the role of the timer genes in *Nasonia* segmentation, we performed embryonic RNAi (eRNAi) against *Nv-opa*. We identified two phenotypes following *opa* eRNAi: a total lack of segments within the embryo and apparent defects in head formation.

To distinguish developmental arrest prior to morphological segmentation from segmentation defects, we used DIC imaging to identify two morphological markers that appear after segmentation in wild-type embryos: presence of the gut wall and (where visible) gonads (Bull, 1982). Surviving embryos (8/67 *egfp-*, 32/64 *opa-*) were scored into six classes. Some embryos had an obvious gut, no gut wall and no segments. These embryos were only present after injection with *opa* dsRNA, and could represent either developmental arrest at a stage prior to morphological segmentation or a segmentation defect. Many embryos successfully formed the gut wall, which forms after morphological segmentation (Bull, 1982), but lacked any distinguishable morphological segments. Some of these embryos also lacked a head.

A small number of *egfp* and *opa* injected embryos had defects in the number of segments. These embryos also exhibited extensive cytoplasmic leakage, suggesting that this phenotype was caused by the injection procedure itself. Completely asegmental embryos occurred in 69% of surviving *opa^−^* embryos and never occurred in *egfp^−^* embryos. Together, these data show that *Nv-opa* is required for morphological segmentation in *Nasonia*, supporting its proposed role as a key regulator of segmentation. The defects in head formation are consistent with the expression of *Nv-opa* in the head (Fig. 3A-E), and its requirement for head formation in *Tribolium* (Clark and Andrew, 2018).

**Fig. 4.**
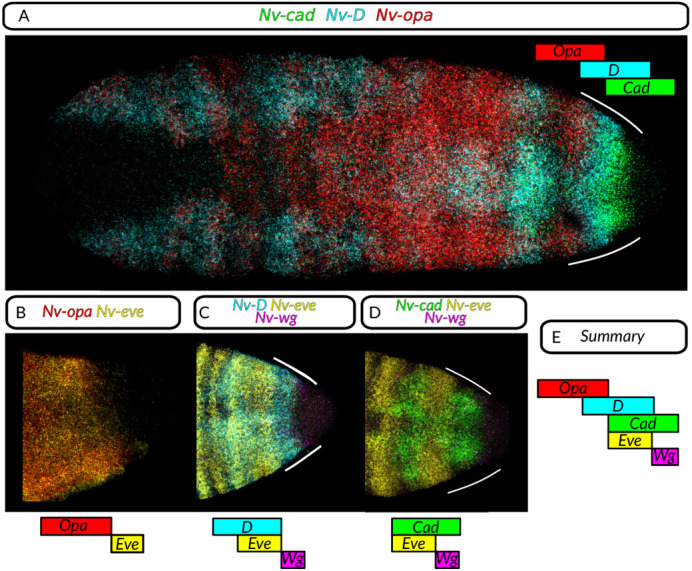
**Timer gene expression in the posterior of the embryo is similar to that in other sequentially segmenting species with overlapping but spatially sequential domains of expression along the A/P axis.** All embryos are partial or full maximum intensity projections, anterior leftwards. (A) Lateral view; whole embryo stained for *cad*, *opa* and *D*. (B) Partial embryo; lateral view; stained for *opa* and *eve*. (C) Posterior; dorsal view; stained for *D*, *eve* and *wg*. (D) Posterior; ventral view; stained for *eve*, *cad* and *wg*. (E) Schematic describing gene expression in the posterior.

We then investigated the sequential appearance of stripe 15, again by staining for timer gene expression. Before stripe 15 appearance, and from stage iii, *Nv-cad* and *Nv-D* RNA are expressed in overlapping stripes behind the sixth *eve* stripe, with *Nv-cad* most posterior and overlapping with the *Nv-wg* domain (Figs 3E and 4C). Later, in stage vii, the 15th *eve* stripe appears within the *Nv-cad*/*Nv-D* domain, anterior to the posterior *Nv-wg* stripe (Fig. 4C-F).

At this stage, a posterior *Nv-opa* stripe is visible within the 6th *eve* stripe. *Nv-opa* is expressed up to the anterior end of the 15th *eve* stripe (Fig. 4D). At stage viii, the *eve* stripe is still expressed within the *Nv-D* and *Nv-cad* domain, but *Nv-opa* has expanded posteriorly to be co-expressed with *eve* at the anterior of the *eve* stripe. Thus, *Nasonia* possess a similar spatial sequence of the timer genes as *Tribolium* and *Drosophila* stripe 15: a spatial sequence of gene expression in the order *Nv-wg→Nv-cad→Nv-D*→*Nv-opa* (posterior to anterior). Whether the *Nasonia* posterior is patterned by an oscillatory GRN is unclear, and our data do not address this. The kinematic waves of *odd* in the posterior argue for an oscillator (Rosenberg et al., 2014).

With the exception of stripe 6, the *Drosophila* GRN combined with the *Nasonia cad* and *opa* expression patterns are able to recapitulate the *Nasonia* dynamics – both the existence of progressive segmentation in the anterior and sequential segmentation in the posterior. However, this analysis relies on two crucial assumptions. First, it assumes that the *Nasonia* genome contains two different pair-rule GRNs. These GRNs are activated by different sets of timer genes and have qualitatively different activity-functions: oscillatory versus non-oscillatory expression of genes. Second, it assumes that the pair-rule networks of *Nasonia* and *Drosophila* are reasonably similar. In the following sections, we use our description of *Nasonia* segmentation to address both these points.

### *Nasonia* pair-rule genes are regulated by two gene regulatory networks

Assessing whether one or two GRNs act in a given process is challenging. We observe, however, startling coordination between structural and behavioural changes in gene expression, which we interpret as meaning that two GRNs are acting in *Nasonia* segmentation. Frequency doubling (of *Nv-slp* and *Nv-eve*) and expression of segment polarity genes begins at stage iv, and occurs in an anterior to posterior progression within the embryo (Fig. 1).

Segmental expression of *Nv-prd* also begins at this stage, and again occurs in an anterior to posterior progression, with segmental expression of *Nv-prd* being detectable at the same time as *Nv-eve* and *Nv-slp* frequency doubling (Fig. 1). There is also a dramatic shift in the relative expression of *Nv-slp* and *Nv-eve*: these genes change from being co-expressed to being strongly anti-correlated within the embryo, implying that the regulatory relationship between them has changed (see Fig. 7). These changes are tightly coordinated throughout the embryo, implying that they share a common cause. Expression of *Nv-opa* precedes these changes, implying that *Nv-opa* may cause these changes (Fig. 3), an observation strengthened by the fact that *Nv-opa* is required for morphological segmentation. We also observe a change in stripe dynamics at stage iv. The second and third pair-rule stripes shift forwards until stage iv or v, then stop (Fig. 2A). This change in gene expression dynamics implies that the activity-function of the networks underpinning these gene expression patterns has changed. Together, these data imply that *Nasonia* possess two pair-rule GRNs [or functional/pragmatic modules of a larger pair-rule GRN (Verd et al., 2019)] with two distinct activity-functions.

### The early segmentation gene regulatory network is conserved between *Nasonia* and *Drosophila*

**Fig. 5.**
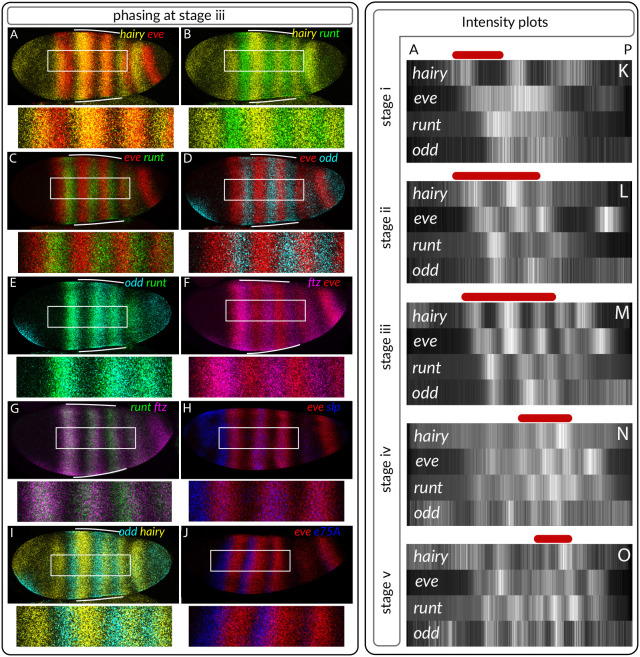
**Phasing of the *Nasonia* early network closely resembles that of *Drosophila*.** (A-J) Maximum intensity projections of embryos stained for pair-rule genes. All embryos are laterally oriented, anterior left. White bars indicate the stripes that have the pair-rule pattern established. (K-O) Intensity plots describing gene expression at different embryo stages. Signal is averaged from 10-50 µm along a line following the curvature of the embryo, and are normalised 0 and 1. Background is defined as the signal intensity present in the head where only *hairy* is expressed. White bars in A-J indicate high intensity of gene expression. A, anterior; P, posterior. Red bars in K-O indicate the region of the embryo with the pair-rule pattern established. Stage iv and v embryos are beginning to undergo gastrulation so anterior stripes are more disordered.

We then investigated the conservation of the two pair-rule networks we have identified, comparing these with *Drosophila*. In the anterior, the initial pattern of the *Nasonia* early network is specified by gap inputs (Rosenberg et al., 2014). The *Nasonia* primary pair-rule genes are expressed in the same order as *Drosophila*, *Nv-hairy*→*Nv-eve*→*Nv-runt*→*Nv-odd*/*Nv-ftz*→*Nv-hairy* (see Fig. 5A-J). Also like *Drosophila*, these stripes move anteriorly over time (Fig. 2). A key difference arises in the expression of the secondary pair-rule genes: *Nv-slp* and *Nv-prd*. *Nv-slp* RNA is expressed within, not between, *Nv-eve* RNA stripes (Fig. 5C), and *Nv-Prd* RNA is not expressed until later in segmentation (Fig. 1). In addition, we identified early pair-rule expression of *Nv-E75a* RNA anterior to *eve* RNA (Fig. 5D), implying that this gene may also be involved in early segmentation.

In line with the time delay in timer gene expression along the AP axis, this primary pair-rule pattern matures over time. To concisely visualise this, we quantified gene expression along the midline of the embryo (averaging across 10-50 µm), and plotted these intensities as colour gradients. The *hairy*-*eve*-*runt*-*odd* pattern develops over time, from being present only in the very anterior at stage i, to being present in the fourth and fifth stripes at stage iv (Fig. 5K-O).

In the posterior, gene expression begins with a cap of *Nv-odd* expression (Rosenberg et al., 2014, Fig. 1). At stage iv, a posterior stripe of *Nv-runt* forms anterior to this *Nv-odd* cap. A stripe of *Nv-hairy* then subdivides the *Nv-odd* cap, and the 15th *eve* stripe is expressed (Fig. S8). This produces a spatial pair-rule gene expression sequence (anterior to posterior), *Nv-runt*→*Nv-odd*→*Nv-hairy*→*Nv-eve*, as well as a temporal *Nv-odd*→*Nv-hairy*→*Nv-eve* sequence in the region of the 15th *eve* stripe, again consistent with the expression sequence from *Drosophila*. However, no cell goes though the full sequence of gene expression.

The relative timing of gene expression is similar throughout the axis. *Nv-hairy* is the first gene to be expressed in a pair-rule manner, followed by *Nv-odd*, *Nv-runt* and *Nv-eve*. This implies that: (1) a similar patterning process is acting in different regions of the embryo, just at different times; and (2) that *Nv-hairy* and *Nv-odd* play an important role in establishing initial pair-rule pattern. This could be further tested by eRNAi followed by analysis of gene expression patterns.

**Fig. 6.**
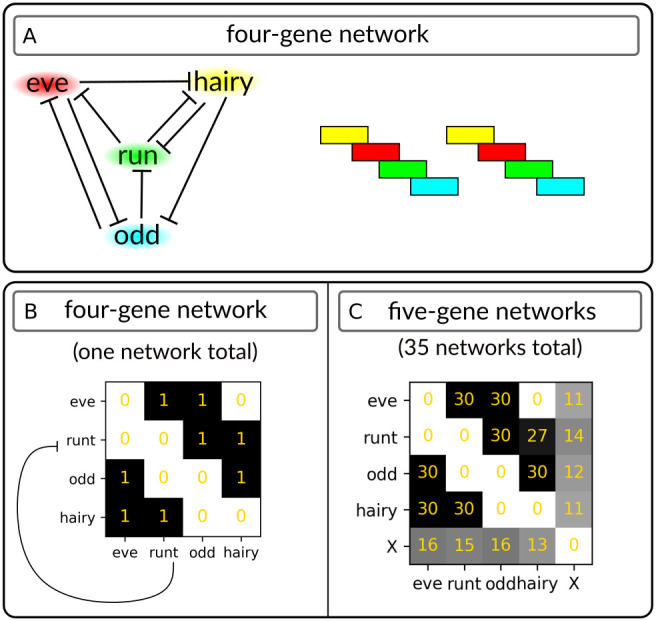
**Networks capable of the observed gene expression patterns and sequential patterning.** (A) Four-gene network topology (left) and associated gene phasing (right). (B) Topology of the four-gene network visualised as a matrix. Repressive interactions are read *x* to *y*, i.e. *runt* represses *eve*. (C) Five-gene networks possessing a given genetic interaction. Frequency of interaction is given in gold. Gene X: unconstrained fifth gene initialised with the same expression as *hairy*.

To what extent does such similarity in gene expression patterns imply topological similarity between the *Nasonia* and *Drosophila* networks? There must be topological changes to secondary pair-rule gene regulation in *Nasonia*, as the expression of these genes is inconsistent with the *Drosophila* topology. Other pair-rule patterns could be trivially modelled using the *Drosophila* network (Clark, 2017), as these patterns are identical. However, we wished to know to what extent these particular gene phasings constrain sequentially segmenting network topologies. Accordingly, to identify a four-gene topology capable of sequential segmentation and the *Nasonia* phasings, we performed a computational screen. Topologies were classed as successful if, after 100 time-points of the simulation (about 2.5 full oscillations), they went through the sequence *hairy*→*hairy/eve*→*eve*→*eve/runt*→*runt*→*runt/odd*→*odd*→*odd/hairy*→*hairy*. This screen identified one topology capable of producing sequential segmentation from normal inputs – the topology in Fig. 6A,B. Thus, the gene expression sequence observed in *Nasonia* and *Drosophila* is consistent with sequential patterning, although this sequence does constrain the possible topology of the early network. Unlike the hypothetical network of Clark (2017), our potential network requires the positions of every pair-rule gene to be provided to the simulation in order to produce normal patterning (Fig. S4), meaning that simultaneous patterning using this topology requires an extensive (presumably gap gene-mediated) spatial pre-pattern. We wished to know whether adding extra genes (representing patterning by gap genes or other pair-rule genes) confers additional flexibility to the network. We screened for five-gene topologies capable of sequential segmentation, with the fifth gene (gene X) initially co-expressed with *hairy* but with no final restrictions on its expression. After filtering out networks with topologies identical to the four-gene network, this analysis identified 35 potential networks, for which the frequency of genetic interactions are presented in Fig. 6C. Although some genetic interactions remained impossible for this model formulation, the addition of the extra gene provided flexibility to the network, ensuring that no genetic interaction was present in every predicted network. Taken together, this analysis shows that the *Nasonia* network could be capable of oscillatory gene expression and sequential segmentation.

### Topological changes are required to explain some aspects of late *Nasonia* gene expression

**Fig. 7.**
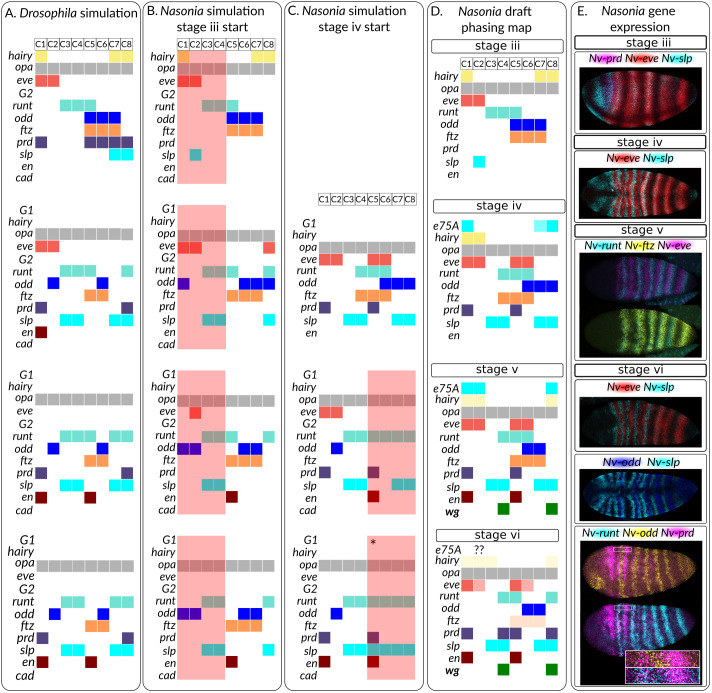
**Altered inputs can recapitulate some aspects of late *Nasonia* segmentation.** Each cell takes different numbers of time-steps to reach final output states, so the temporal information depicted is not necessarily accurate. (A) *Drosophila* simulations based on gene expression at *t*=36 timepoint (Clark, 2017), i.e. similar to *Nasonia* except for *prd* and *slp*. In cell 1 (C1), *slp* expression was omitted because this gene would decay without regulatory input. (B) *Nasonia* simulations based on expression at stage iii. Red background indicates regions where proper patterning is not produced. (C) *Nasonia* simulations, starting with gene expression at stage iv. C4 (asterisk) becomes an attractor cycle because of incorrect eve regulation. (D) Draft map of gene expression in the *Nasonia* embryo. Map is based on gene expression in the first four segments, which is representative of expression throughout the embryo. The map is produced by comparisons to *Nv-eve* and *Nv-wg*, so other gene-gene comparisons may be less accurate. (E) Selected *in-situ* hybridisation stains showing relative expression of genes. All embryos are maximum intensity projections, anterior leftwards. See Figs S6-S8 for the rest of the dataset.

Changes to the *Nasonia* late gene expression, relative to *Drosophila*, are as follows. Only *Nv-eve*, *Nv-slp* and *Nv-runt* undergo frequency doubling, whereas in *Drosophila*, *Dm-odd* also doubles (Schroeder et al., 2011; Rosenberg et al., 2014). *Nv-odd* is expressed as a two-cell wide stripe, not one-cell wide, while secondary *Nv-runt* stripes are expressed very briefly and are only one-cell wide (Fig. 1, Rosenberg et al., 2014). Our positioning of the *runt* stripes disagrees with that of Rosenberg et al. (2014): we place the strong *runt* stripe in the even-numbered parasegment (Fig. 7D,E). Additionally, *Nv-slp* and *Nv-odd* overlap (Fig. 7E). We wished to know whether the observed changes in expression in the late network could be explained by changes in input to the late network – i.e. the altered positioning of *Nv-slp*, *Nv-prd* and *Nv-E75A* – or whether they require changes in gene regulation.

To approach this problem, we used an R package, BoolNet (Müssel et al., 2010), to model the output of the *Drosophila* network under various input conditions. To avoid arbitrarily assigning protein and RNA ages when initialising models, we ignored the gap between RNA transcription and protein translation, unlike Clark's model (Clark, 2017). Modelling *Drosophila* gene expression at the t=36 timepoint produces gene expression patterns identical to the existing model if *slp* expression in cell 1 is ignored (this expression would decay naturally) (Fig. 7A).

Modelling the *Nasonia* stage iii gene expression was able to produce some aspects of *Nasonia* like patterning of the even-numbered parasegment. In this segment, the main difference between *Drosophila* and *Nasonia* is that *odd* stripes are two cells wide, and that these cells co-express *slp* (Fig. 7D). Additionally, towards the end of segmentation, *Nv-runt* is expressed in one-cell wide stripes, co-expressed with *Nv-wg*. The altered inputs to the even numbered parasegments are able to recapitulate these patterns: the lack of *slp* expression posterior to *odd* means that the *odd* and *ftz* domains are not repressed posteriorly, and these genes remain as two or three cell stripes. In these simulated cells, *slp* and *prd* expression do not resolve properly. *slp* fails to resolve because, in *Nasonia*, *slp* and *odd* are co-expressed, a pattern inconsistent with the mutual repression of these genes in the *Drosophila* model used. *prd* never becomes expressed because *prd* expression is only possible in cells already expressing *prd* and we supply no *Nv-prd* expression to the model. As in the *Drosophila* model, secondary *eve* stripes do not appear. Thus, the *Nasonia* inputs to the *Drosophila* network are able to produce some of the altered *Nasonia* gene expression.

We were unable to produce proper patterning in odd-numbered parasegments. Secondary *runt* and *odd* stripes form, which are not present in *Nasonia* embryos, and *odd* is mis-positioned. To check that this was not due to the lack of *prd*, we initialised the model with *Nasonia* inputs from stage iv. This produced *Drosophila*, not *Nasonia*, gene expression patterns, and incorrect phasings in C4-C8 (compare Fig. 7A-C). Thus, the *Drosophila* model cannot produce the *Nasonia* odd-numbered stripe gene expression. Involvement of an additional gene is required to produce these changes. In the *Nasonia* cell expressing *odd*, *eve* is the only gene to be expressed, and so is the only modelled pair-rule gene that could turn *odd* off in this cell (Fig. 7B-D). However, *Nv-odd* and *Nv-eve* are stably co-expressed, so cannot repress each other (Fig. 7D). Thus, additional genes must be involved to repress *odd* in this cell. A similar argument holds for *runt*: in cells 3 and 4, only *slp* is co-expressed with *runt* so only *slp* could repress *runt*. Again, there cannot be a repressive interaction between these genes, because *slp* is co-expressed with *runt* in cell 8. This analysis assumes similar production and degradation rates for all genes: if this does not hold, then these predictions will not either.

In summary, the *Drosophila* network can produce the expanded *odd* and *ftz* expression in *Nasonia*, and the smaller *runt* domains, but cannot recapitulate the lack of *odd* doubling and the general patterning in cells 1-4. Evolutionary changes in gene regulation are required to explain these features.

## DISCUSSION

Here, we explore the conservation of the pair-rule GRN in *Nasonia* and *Drosophila*. We found that altered inputs to a largely conserved *Drosophila* network can explain the difference between *Nasonia* and *Drosophila* pair-rule patterning. The general organisation of interactions between the pair-rule genes into two networks is likely conserved in *Nasonia*. In addition, although there are changes to patterns of gene expression in the late network, the early network still appears to behave in a similar way to *Drosophila*. We were able to recapitulate the *Nasonia* segmentation dynamics – progressive patterning – using the *Nasonia* timer gene expression patterns. Finally, changes in the input to the late network are able to recapitulate some, but not all, changes in *Nasonia* segmentation, implying some regulatory changes in the late network.

### The timer gene hypothesis explains the dynamics of *Nasonia* segmentation

We are able to model and explain the broad strokes of *Nasonia* segmentation – its progressive and sequential patterning – using the timer gene hypothesis. Anterior expression of timer genes was used to model progressive patterning, while in the posterior, the timer genes are expressed in a similar spatial sequence to the other sequentially segmenting insect studied, *Tribolium* (Clark and Andrew, 2018). Presumably, the GRN used does not change between the anterior and posterior of *Nasonia*. Moreover, the apparent separation of *Nasonia* patterning into two GRNs provides support for the timer gene idea, which relies on two GRNs with different behaviours being activated in different ways, to achieve patterning (Clark, 2017). This provides explicit quantitative support for the timer gene hypothesis.

**Fig. 8.**
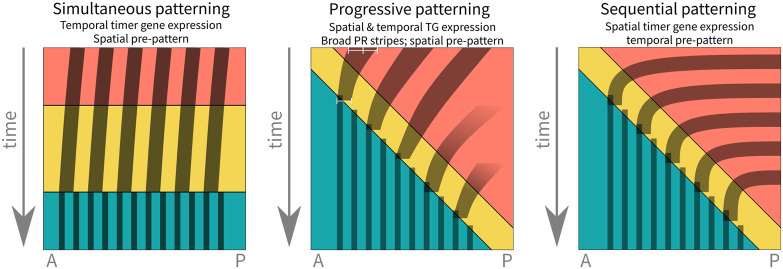
**Schematic of simultaneous, sequential and progressive segmentation.** Adapted, with permission, from Clark et al. (2019).

Additionally, inspired by *Nasonia* patterning, we provide a way to combine a spatial and temporal pre-pattern, producing what we have called progressive patterning. Broad pair-rule stripes (an expanded spatial pattern) correct for the temporal pattern, e.g. an expansion/retraction of *cad* and *opa* expression, ultimately leading to progressive patterning (Fig. 8).

This finding has important implications for the evolution of segmentation. It means that full simultaneous and sequential segmentation are likely two ends of a spectrum, rather than distinct types of segmentation. Such a spectrum is implied in the findings of Clark (2017) and Clark and Andrew (2018): if simultaneous and sequential segmentation share a mechanistic basis or GRN, then intermediates could exist. Small changes to the expression of the timer genes, and the width of pair-rule stripes, can have a dramatic impact on how the pair-rule GRN behaves, and so to the dynamics of segmentation, providing a simple and elegant method of evolving phenotypic variation.

### How do we define GRNs as similar or different?

Assessing whether two GRNs are the same is an interesting and unsolved problem. Using structural similarity does not work (DiFrisco and Johannes, 2019). We instead take an established model and/or set of hypotheses and identify features of the network that are crucial to its function – either necessary or emergent properties of the network. The gene regulatory process could be the same or different in these regards.

We think that the *Nasonia* network is the same as that of *Drosophila* with regards to the primary pair-rule genes. These are expressed in the same order as in *Drosophila*, exhibit similar dynamics (anterior stripe shifts) and, crucially, differences in their dynamics (progressive patterning) can be explained using the *Drosophila* model.

The networks are different with regards to the secondary pair-rule genes and the switch from pair-rule to single segment periodicity: *Nv-prd* and *Nv-slp* are expressed differently to their *Drosophila* counterparts. Moreover, the co-expression of *Nv-slp* and *Nv-eve* implies regulatory evolution. In this way, *Nasonia* resemble *Tribolium*, not *Drosophila*, where the even-numbered stripe develops first (Choe and Susan, 2007). These differences can explain some, but not all, aspects of the late network gene expression in *Nasonia*, implying that there are changes to the late network topology. For example, the expanded *odd* and *ftz* domains in *Nasonia* can be explained by the *Drosophila* network and by the altered inputs of *Nasonia* to it, while the lack of *odd* frequency doubling requires topological changes to the network. We are able to predict some of these changes. First, *Nasonia* must lack strong mutual repression between *slp* and *odd* to maintain stable co-expression of these genes. Second, there must be another gene or genes patterning the anterior parasegment (cells 1-4), to prevent frequency doubling of *Nv-runt* and *Nv-odd*. Third, the regulation of *Nv-eve* must change to allow *eve* frequency doubling. Additionally, the pair-rule expression of *Nv-e75A* implies that this gene may be required for segmentation in *Nasonia*, the first evidence of its use in pair-rule patterning outside true bugs (Erezyilmaz et al., 2009; Reding et al., 2019; Hernandez et al., 2020).

The output of the late network, expression of the segment polarity genes, is still largely identical to *Drosophila*, consistent with previous empirical and modelling work showing the segment polarity network is stable and well conserved (Von Dassow and Eil, 2004; Choe and Susan, 2009; Janssen and Graham, 2013; Green and Michael, 2013; Vellutini and Andreas, 2016; Auman and Ariel, 2018; Clark et al., 2019) (Fig. S2). We interpret this as meaning the activity-function of the late network, in this case the output/attractors of the system, is unchanged.

We would like to know whether the *Nasonia* and *Drosophila* networks are homologous. We cannot assess structural homology, as argued above. We instead argue that the *Nasonia* and *Drosophila* GRNs are functionally homologous: they share an activity-function (dynamic behaviour of the primary pair-rule genes and relative expression of the segment polarity genes), but differ in use-function (progressive versus simultaneous patterning) (Love, 2007). They also share common descent: the involvement of pair-rule genes in arthropod segmentation is extensively documented, and is at least as old as holometabolous insects (Dearden et al., 2006) and likely older (Dearden et al., 2002; Damen et al., 2000; Green and Michael, 2013). It is unclear whether these GRNs are structurally homologous, as we collected no data on the interactions between *Nasonia* pair-rule genes. Although there are changes to gene regulation in the late network, the output of segmentation is the same (expression of the segment polarity genes), and there is no evidence for a change in the dynamics of stripe expression (e.g. anterior movement of stripes). We think that this conservation of output and behaviour means that the function of the late network is unchanged. Thus, the *Drosophila* and *Nasonia* GRNs may be thought of as functionally homologous (Love, 2007; DiFrisco and Johannes, 2021), despite 300 million years of evolutionary divergence.

### Studying GRNs

These findings reinforce a number of important points. The behaviours of the *Drosophila* and *Nasonia* pair-rule networks can only be understood using dynamical modelling (Clark, 2017; Figs 5 and 3), reinforcing the necessity of using modelling as a tool to understand GRNs (DiFrisco and Johannes, 2019; Briscoe, 2019). Second, the late GRN is input dependent: changes to the inputs of this GRN are able to explain some (but not all) changes in late *Nasonia* gene expression. Again, this reinforces the need to use modelling alongside empirical research, and in particular the power of using modelling to understand developmental processes in less-studied species such as *Nasonia*.

### Conclusions

Overall, we find that remarkably few changes to the *Drosophila* pair-rule GRN are required to simulate *Nasonia* patterning. The progressive patterning of *Nasonia* can be recapitulated by changing the *Nv-cad* and *Nv-opa* dynamics, while some changes to the late network can be simulated using only the changed *slp* and *prd* expression at stage iii. Our method gives no direct evidence that specific interactions in the *Drosophila* GRN are conserved in *Nasonia*, but does imply that if there are substantial topological changes to the *Nasonia* network, these do not result in changes to the patterning process.

Finally, the similarities between *Nasonia* and *Drosophila* segmentation, at the level of the GRN involved, imply that these derive from a common ancestor GRN that likely evolved deep in the arthropod lineage. This GRN has proven developmentally flexible over evolutionary time, allowing different forms of morphological segmentation to be built on an overall conservative network. The changes we have detected in the GRN that underlie *Nasonia* segmentation are limited, implying that only minor modifications of an ancestral but flexible GRN may be enough to generate wide variety in morphological segmentation.

## MATERIALS AND METHODS

### Hybridisation chain reaction

*Nasonia* were raised on commercially sourced *Sarcophaga bullata* pupae (from Mantis Mayhem, https://mantismayhem.co.uk/shop/ols/products/green-blue-bottle-flies-pupae), at 25°C. *Nasonia* cultures were a kind gift from Dr David Shuker and Dr Nicola Cook (St Andrews University, UK).

Embryo collection was carried out according to Werren and David (2009). Plugs were modified such that *S. bullata* pupae could be placed, head-exposed, inside the plug. Embryos were collected from pupae provided to *Nasonia* for 12 h, to provide the relevant stages of segmentation. Some embryos were stored in the fridge overnight for convenience. Hosts were cracked open under a dissecting microscope and dipped into 5 ml of heptane, 4.5 ml of PBS (phosphate-buffered saline) and 0.5 ml 37% formaldehyde in a 15 ml Falcon tube, and fixed for 8-18 h. After fixing, embryos collected at the bottom of the Falcon tube. To dechorionate the embryos, the bottom formaldehyde layer was replaced with 100% ice-cold methanol and shaken vigorously for 1-3 min. Dechorionated and devitellenised embryos settled at the bottom of the Falcon tube and were transferred to an Eppendorf tube, washed three times in methanol and stored at −20°C.

Hybridisation chain reaction was performed according to Choi et al. (2016). Proteinase K digestion was not required for efficient probe penetration, so embryos were dehydrated in a methanol series before incubation in 100 µl hybridisation buffer for 30 min at 37°C. 1 pmol probe (i.e. 1 µl of 1 µM probe) was added to 50 µl hybridisation buffer, and embryos were incubated with probe overnight at 37°C. Embryos were then washed four times with probe wash buffer for 15 min each at 37°C, before being washed three times for 5 min each in 5×SSCT (5×sodium citrate, 0.1% Tween-20 buffer). Embryos were pre-amplified in amplification buffer for 30 min. Hairpins were prepared by heating each hairpin individually to 95°C for 90 s, and leaving in a drawer at room temperature for 30 min. Hairpin (3 pmol) was added to 50 µl of amplification buffer and added to the embryos. Embryos were amplified overnight in the dark at room temperature. Embryos were then washed twice for 5 min, twice for 30 min and once for 5 min in SSCT. DAPI (Invitrogen) was added to the first 30 min wash. Embryos were mounted in ProLong Glass (Invitrogen), left to cure at room temperature overnight and stored in the fridge before imaging.

Imaging was performed using a Olympus FV3000 confocal microscope at the Department of Zoology, Cambridge University, UK using the UPLSAPO30X 30X silicon oil lens (numerical aperture=1.406). We used Alexa488, Alexa546, Alexa594 and Alexa647-conjugated hairpins from Molecular Instruments. We used 405, 488, 561, 594 and 640 nm lasers, using narrow emission collection windows where necessary to eliminate bleed-through between close fluorophores (Alexa546 and 594).

### Modelling

All scripts are available at https://github.com/Shannon-E-Taylor/masters. Simulations of the pair-rule system were performed using the modelling framework described by Clark (2017), and the code from the supplemental information of that paper. The model consists of a one-dimensional row of cells. Boolean network analysis was performed using the BoolNet R (R version 3.4.4) package (R Core Team, 2018; Müssel et al., 2010). The full GRN from the supplemental information of Clark (2017) was used for modelling. To generate a state graph, the plotStateGraph function was used. To identify attractors, the getAttractors function was used; the default version of this code identifies all attractors for a synchronous network using the exhaustive method, which identifies trajectories for every possible initial condition.

### Image analysis

Image analysis was carried out using Fiji (Abràmoff et al., 2004; Schindelin et al., 2012). Analysis of blastoderm-stage embryos was carried out on partial or full maximum intensity projections; where embryos had more than one cell layer, relationships between genes were confirmed using the original 3D images. Background fluorescence was defined as the fluorescence visible in regions of the embryo clearly not expressing the gene of interest (for most embryos, the head) and was removed using the brightness-contrast tool in Fiji.

### Embryonic RNA interference

Embryonic RNAi (eRNAi) was performed in Aotearoa, New Zealand using a different strain of wasps from those in Cambridge, UK. These *Nasonia* were reared on *Lucilia sericata* blowflies (www.biosuppliers.com), using similar methods to those of Werren and David (2009) (*S. bullata* are not commercially available in Aotearoa). dsRNA against *egfp* and *Nv-opa* was ordered from Genolution (http://genolution.co.kr/agrorna/service-overview/).

To prepare embryos for microinjection, adult wasps were exposed to hosts for 2-5 h. This long exposure time led to embryos of very similar stages, because the *Nasonia* took several hours to prepare to lay. Embryos were gently collected using fine forceps and aligned on a 1% agarose/PBS plate. Embryos were affixed to coverslips using heptane glue and covered with a small drop of *Drosophila* microinjection oil (1.75 ml Halocarbon oil 700+0.25 ml Halocarbon oil 27, Sigma). For the experiment reported here, embryos were then left at 4°C for 4 h until it was time to inject them; these embryos were blastoderm stage embryos prior to cellularisation. Other experiments, injecting embryos at the time of pole cell formation, resulted in similar phenotypes.

Embryos were injected using a PLI-100 (Harvard Apparatus) injection apparatus, and borosilicate needles. The coverslip was then transferred to the agarose plate to maintain humidity, and incubated at 28°C until all embryos had fully developed (24-36 h). Imaging was performed using DIC optics and an Olympus BX61 compound microscope.

## Supplementary Material

Supplementary information

Reviewer comments
