# Supplementary material for: The Nasonia pair-rule gene regulatory network retains its function over 300 million years of evolution
Source: Development. 2022 Mar 9;149(5):dev199632. doi: 10.1242/dev.199632 (PMC8959145; doi:10.1242/dev.199632)
Supplement: Reviewer comments [file develop-149-199632_review_history.pdf]

The *Nasonia* pair rule gene regulatory network retains its function over 300 million years of evolution

Shannon E. Taylor and Peter K. Dearden
DOI: 10.1242/dev.199632

Editor: Cassandra Extavour

Review timeline

Original submission:	4 May 2021
Editorial decision:	23 July 2021
First revision received:	30 September 2021
Editorial decision:	16 December 2021
Second revision received:	19 January 2022
Accepted:	28 January 2022

Original submission

First decision letter

MS ID#: DEVELOP/2021/199632

MS TITLE: Nasonia segmentation is regulated by an ancestral insect segmentation regulatory network also present in flies

AUTHORS: Shannon Taylor and Peter Dearden

I have now received all the referees' reports on the above manuscript, and have reached a decision. The referees' comments are appended below, or you can access them online: please go to BenchPress and click on the 'Manuscripts with Decisions' queue in the Author Area.

As you will see, the referees express considerable interest in your work, but have some significant criticisms and recommend a substantial revision of your manuscript before we can consider publication. If you are able to revise the manuscript along the lines suggested, which may involve further experiments, I will be happy receive a revised version of the manuscript. Your revised paper will be re-reviewed by one or more of the original referees, and acceptance of your manuscript will depend on your addressing satisfactorily the reviewers' major concerns. Please also note that Development will normally permit only one round of major revision.

You will see that Reviewer 2 has a number of suggestions that would involve significant restructuring of the manuscript. In my view, major changes to the structure are not needed unless you find them to be beneficial, and I do not agree with this reviewer that removal or reduction of discussion of the modelling aspect of the paper, from the main text, would be an improvement. In my view, this part of the analysis is one of the major strengths of the work. However, I do think that their suggestion to include more background information in the introduction, and to include less information per figure to make the easier to follow, are useful and would improve the manuscript for readers.

We are aware that you may be experiencing disruption to the normal running of your lab that make experimental revisions challenging. If it would be helpful, we encourage you to contact us to discuss your revision in greater detail. Please send us a point-by-point response indicating where you are able to address concerns raised (either experimentally or by changes to the text) and

© 2022. Published by The Company of Biologists under the terms of the Creative Commons Attribution License (https://creativecommons.org/licenses/by/4.0/).

where you will not be able to do so within the normal timeframe of a revision. We will then provide further guidance. Please also note that we are happy to extend revision timeframes as necessary.

Please attend to all of the reviewers' comments and ensure that you clearly highlight all changes made in the revised manuscript. Please avoid using 'Tracked changes' in Word files as these are lost in PDF conversion. I should be grateful if you would also provide a point-by-point response detailing how you have dealt with the points raised by the reviewers in the 'Response to Reviewers' box. If you do not agree with any of their criticisms or suggestions please explain clearly why this is so.

Reviewer 1

Advance summary and potential significance to field

This interesting study of pair rule gene networks tests the hypothesis that pair rule genes could be activated by expression waves of timer gene to produce progressive segmentation as well as simultaneous segmentation. Using modeling and extensive gene expression data combined with RNAi for oddpaired, the authors present a strong argument for conservation of much of the gene network first discovered and detailed in Drosophila. Modelling is crucial to the the study and should lead to the discovery of new players.

That the pair rule network has to phases is not new, but modeling different inputs will require closer analysis of individual gene regulation.

Comments for the author

The experiments and modeling are described in enough detail to clearly understand the hypothesis and conclusions.
Descriptions in figure legends could be a little more complete such they stand alone and don't rely on having read the cited papers.
The writing is clear and concise. A nice read.

Reviewer 2

Advance summary and potential significance to field

This paper analyses the dynamics of segment gene expression in the parasitoid wasp Nasonia vitripennis. Due to its phylogenetic position and mode of development, Nasonia is an exciting model system for understanding the mechanism and evolution of arthropod segmentation: hymenopterans such as Nasonia evolved long germ segmentation independently from dipterans (indeed simultaneously segmenting Drosophila melanogaster and sequentially segmenting Tribolium castaneum are more closely related to each other than either is to Nasonia). Understanding segmentation gene regulation in Nasonia therefore has the potential to teach us much about the structure and evolutionary flexibility of arthropod segment patterning networks.

There is previous work on Nasonia segmentation, for example the Desplan group looked at caudal and gap genes (Olesnicky et al 2006) and later at the pair-rule genes prd (Keller et al 2010), hairy, eve, runt, and odd (Rosenberg et al 2014), using in situ hybridisation and knockdowns. This paper is also focused on the pair-rule genes, but looks at a larger number of genes and uses a newer in situ method, HCR, which enables relative expression boundaries to be determined through multiplex imaging. The paper also uses a Boolean modelling approach to make inferences about the topology of the Nasonia segmentation network, and suggest similarities and differences with pair-rule gene cross-regulation in Drosophila.

There is lots to find interesting in this paper, although the organisation of the text and the presentation of the figures will need to be significantly reworked before it is ready for publication (see below). In my view, the key contributions of the paper to the broader field are the following:

© 2022. Published by The Company of Biologists under the terms of the Creative Commons Attribution License (https://creativecommons.org/licenses/by/4.0/).

1) Strong support for the timer gene hypothesis. TFs such as cad, D, and opa, which regulate segmentation genes in Drosophila, have been proposed to control the timing of segmentation across arthropods/insects with divergent modes of segmentation, such as Drosophila and Tribolium (eg Clark and Peel Development 2018). Nasonia has segmentation dynamics that are different from both Drosophila and Tribolium, but again, the timer genes are expressed in exactly the way that would be predicted if they play this role, and the functional data from opa RNAi supports this interpretation as well. (Nv-cad expression has been previously analysed but I believe the descriptions of Nv-D and Nv-opa are new.)

2) Documentation of and functional insight into Nasonia stripe dynamics, again supporting the timer gene hypothesis. As was inferable from previous Nasonia (and Apis) papers, but is here explicitly quantitated, the pair-rule pattern shifts anteriorly and compresses significantly over time. The authors show by simulation that this pattern compression is exactly what is expected to happen if tissue maturation is under control of a moving wavefront (rather than a ubiquitous timer), as suggested by the expression of the timer genes. [This mechanism is not specific to pair-rule genes or arthropods, and could be recast in the more general clock-and-wavefront models used by the vertebrate somitogenesis field.] Nasonia is therefore a very neat representation of an intermediate between Tribolium-type and Drosophila-type segmentation, exemplifying how the extreme Drosophila-type mode might have evolved. The wavefront/compression mechanism could also be highlighted as a way an embryo might use time to derive a fine-scale spatial output from a coarser initial pattern.

3) A fuller picture about patterns of conservation and divergence in arthropod segmentation networks. PRG patterns have been catalogued in various species, but detailed descriptions of how relative boundaries resolve over time are quite rare. The data here help flesh out an emerging comparative picture: 1) secondary PRG and segment-polarity gene expression at the parasegment boundary (Fig S2) is strongly conserved, as seen across diverse species; 2) the early pair-rule pattern (ie the phasing of the primary PRGs) also seems quite strongly conserved, although interestingly the inclusion of ecdysone pathway-related genes such as ftz, ftz-f1 and e75a varies across insects; 3) the way that the pair-rule pattern resolves to single-segment periodicity is quite variable across insects, with evidence of changes to the regulation of both the secondary pair-rule genes and late primary pair-rule gene expression. Understanding this last aspect of network evolution seems necessary for understanding how pair-rule patterning (double segment periodicity) evolved within arthropods, which is one of the big open questions in the field.

Comments for the author

For the text, I would recommend that the authors think about how best to walk readers through a topic which can seem dauntingly complex, including providing sufficient background context to understand the findings, and properly emphasising the evolutionary significance of the key results. Some of the less essential results, such as some of the model analysis, could be moved to the supplementary information, to maintain the flow of the text.
More specifically, it would be helpful if the introduction could be revised to provide more context about: 1) the phylogenetic position of Nasonia and the distribution of different modes of segmentation among arthropods; 2) previous work on Nasonia segmentation; 3) an overview of the relevant components of the Drosophila segmentation network (primary PRGs vs secondary PRGs vs segment-polarity genes, early vs late expression patterns and dynamics, timer genes); 4) how these have been proposed to relate to an ancestral clock and wavefront mechanism; 5) key questions to be investigated.
For the results, in my opinion a more logical order of presentation would be to first present all the pair-rule gene expression data, including summary schematics, commenting on 1) the time lag across the AP axis, 2) the anterior shifting and spatial compression of the pattern over time, and 3) the overt similarities and differences with pattern resolution in Drosophila. Then 1) present the timer gene data to provide a mechanistic explanation for the time lag, 2) quantify the shifts/compression and explain the patterning dynamics as a consequence of the timer gene expression using simple simulations, and 3) analyse which aspects of PRG regulation are likely to be conserved / divergent with respect to Drosophila (and potentially other insects), noting how these hypotheses could be functionally tested where necessary.
Throughout, care should be taken to clearly distinguish between 1) the mechanism of periodicity generation, 2) the dynamics of pattern resolution, and 3) time differences in pattern maturation

© 2022. Published by The Company of Biologists under the terms of the Creative Commons Attribution License (https://creativecommons.org/licenses/by/4.0/).

along the AP axis, when discussing modes of segmentation. For the take away message, I think it would be more informative to focus more on the overall pattern of network conservation and divergence (eg regulation by timer genes, primary PRG phasing, parasegment boundary expression all fairly conserved; timer gene axis dynamics and the transition to segmental expression are not) rather than emphasising only the conserved aspects.
I would also recommend that the authors rework and polish the figures, again with the aim of walking the reader through the key results. Overcrowded figures should be split up, layout/colours/labels/annotations optimised for information communication, embryo orientation made more consistent, enlarged image crops used to more clearly communicate stripe phasing etc. I was also surprised that there was not much use made of the multiplexed nature of the image data in the main display figures; this seems a missed opportunity. To improve SNR, the authors might try experimenting with average projection instead of maximum projection, and/or project over a smaller Z range.
The discussion should also be revised in light of the changes made to the rest of the manuscript.
Line-by-line comment on the text follow.
title / abstract –these should be revised to more accurately reflect the content/significance of the paper. (See above.)
line 14 – why these three species in particular?
line 29 – I would argue “extensively” applies only to Drosophila.
line 31 – should Akam 1989 be Akam 1987 (the Development review) instead?
line 33 – there are others as well, such as maternal Hb and the terminal system.
line 42 – that this is a spectrum rather than a dichotomy has been stressed elsewhere, including in the reviews just cited.
lines 46-48 – I would suggest a slightly different definition, i.e. that stripes appear de novo (arguing for periodicity being generated by instructive positional cues rather than segmentation clock), while the tissue / segment pattern matures in an anterior to posterior progression. The periodicity generation and temporal regulation aspects of patterning are therefore contrasted and kept distinct.
line 48 – “Nasonia” is used throughout as a plural. Is this standard?
line 48 – It’s worth being more circumspect here; I don’t think that “sequential” (ie clock-driven) segmentation in the posterior is solidly established. Based on PRG expression dynamics and various knockdown phenotypes, Rosenberg et al 2014 proposed that Nasonia possesses a “dual” mode of segmentation, which is “Drosophila-like” in the anterior (pair-rule stripes patterned by gap genes) and “Tribolium-like” in the posterior (pair-rule stripes patterned by dynamic cross-regulation, by the remnants of segmentation clock). However, although Nasonia stripes do appear (approximately) sequentially, they generally form de novo, arguing against a segmentation clock mechanism. More recently, I have proposed that, even in Drosophila, pair-rule stripe phasing is dynamically established by the remnants of a segmentation clock, meaning that instructive gap gene cues and a large role for pair-rule gene cross-regulation are not mutually exclusive. I think there are at least three separable issues here: 1) how is periodicity established (by gap genes or by a segmentation clock?); 2) what is the rate/time delay of pattern maturation along the tissue (ie how are the timer genes expressed); 3) how important are pair-rule gene expression dynamics in establishing stripe boundaries / phasing (on a spectrum from entirely PRG-regulated to entirely gap gene regulated).
line 53 – the meaning of ‘the same molecular process’ is unclear.
line 54 – it is potentially confusing that here the text talks about an individual instance of segmentation being controlled by two different networks, and earlier about different modes of segmentation being controlled by a single network.
line 65 – Sox21b (not D) is involved in Parasteatoda segmentation.
line 77 – “switch” language doesn’t seem appropriate, since it is previous argued that sequential vs simultaneous timing is a spectrum.
line 79-90 – this section is subsuming a variety of conceptually different things under the heading of “multifunctional GRN”, which I think it would be clearer to distinguish between. First, a single network motif (eg AC/DC) which can give rise to qualitatively different behaviours depending on the quantitative strength of particular component interactions. Second, context-specific gene networks where the regulation of individual genes is strongly dependent on tissue / stage-specific TFs (eg different timer genes activating different pair-rule gene enhancers). Third, a gene network that is deployed in different ways across different tissues / organisms, although its logic and intracellular dynamics are conserved (the pair-rule gene network in different insect species, potentially).

© 2022. Published by The Company of Biologists under the terms of the Creative Commons Attribution License (https://creativecommons.org/licenses/by/4.0/).

line 114 – how was the gene set (including e75a) decided? Which genes have been looked at previously in Nasonia and which are unique to this paper?
line 115 – specify ectoderm / mesoderm boundary?
line 116 – this staging scheme is very confusing, because “eve4” might mean eve stripe 4, or an embryo with 4 eve stripes, none of which is eve4! It would be more intuitive for the reader if eve2 through wg13 were converted to an unified numerical scheme, either in term of time AEL, or just phases/stages 1, 2, …, N.
line 118-163 – it would help the reader if this information was presented one topic at a time. What is the temporal pattern of gene expression in a given region and is this the same across the axis and the same as in other species? How does the timing of gene expression vary along the axis? How does this timing relate to other embryonic events? What are the phasing relationships between the different genes and how do these change over time? Which bits of this information are new in this manuscript vs previously described?
line 145 – see comment about line 48.
line 153-156 – this definition is not straightforward to apply when gastrulation occurs progressively along the AP axis rather than simultaneously. (Note also that the 15th stripe of eve expression forms after gastrulation in Drosophila.)
line 157 – since the PRG stripes resolve over time, it is preferable to quantitate the width of the pair-rule repeat rather than the width of the stripes themselves. (Eg peak to peak or trough to trough.) In Fig 1B, is there a more reliable posterior anchor that can be used instead of eve expression? The posterior border of the final eve stripe shifts quite considerably in Drosophila, for example.
line 165 – it might be more straightforward to talk about the compression of the pattern over time rather than the “expanded initial pattern”.
line 168 – which model?
line 169-172 – the previous description of Nv-cad by Olesnicky et al should be cited.
line 179 – this unusual eve expression should be flagged earlier, when the PRG patterns are described. Channel merges should be included in fig 2 to show the spatial registration of the different patterns.
line 181-189 – this is an important insight, which is complicated somewhat by being presented in the context of the full Drosophila pair-rule model. I would lean towards presenting simpler clock-and-wavefront simulations to explain the point, ie something that looks more like figure 6.
line 190-209 – nice functional data, which should be mentioned in the abstract. It is interesting that the opa knockdown causes an asegmental phenotype, whereas in Drosophila the ftz-dependent boundaries are lost but the prd-dependent boundaries still form. A change in the relative importance of prd and opa for activating segment-polarity gene expression would be consistent with the delayed prd expression seen in Nasonia vs Drosophila.
line 216 – “ventral ends of the embryo” unclear.
line 217-221 – similar spatiotemporal dynamics are seen in the equivalent (tail / eve 15) region of the Drosophila embryo – see eg the supplementary information in Clark and Peel 2018. Think about the colour choices in Fig 3; green/yellow, green/cyan, and red/magenta are all hard to discriminate between.
line 222 – can more be said about what is going on in the stripe 6 region? Exceptions are interesting.
line 226-227 – note that the early Drosophila PRG network involves oscillatory dynamics, but does not involve an intact oscillator per se. It is worth addressing the question of whether Nasonia retains an intact oscillator/segmentation clock in the posterior as has been suggested (I don’t see compelling evidence for this) vs exhibiting oscillatory dynamics which are presumably evolutionary remnants of such an oscillator (there seems to be good evidence for this across most of the axis).
line 230 – I think this section is more complicated than it needs to be. In Drosophila, Opa acts as a pioneer factor at numerous PR and SP gene enhancers (Soluri et al 2020), thereby precipitating about stable, segmental PR and SP gene expression, in part by changing the regulatory interactions between the PRGs (Clark and Akam 2016, Clark 2017). The spatiotemporal dynamics and changing expression relationships (eg eve/slp) of the Nasonia PRGs, combined with the spatiotemporal dynamics of Nv-opa, are consistent with a similar role for Opa being conserved in Nasonia segmentation.
line 246-247 – “topology” or “structure” rather than behaviour?
line 248-249 – see the comment re line 54. It’s probably useful to explicitly address the multiple tissue-specific networks vs single network with subnetworks terminology/usage issue somewhere to avoid confusion.

© 2022. Published by The Company of Biologists under the terms of the Creative Commons Attribution License (https://creativecommons.org/licenses/by/4.0/).

line 255 – runt should be before odd+ftz.
line 255-8 – why colour gradients rather than line plots?
line 258-260 – can anything be inferred from the order in which the stripes of different genes resolve? In Drosophila, the eve pattern resolves before the other pair-rule genes, consistent with eve's extensive regulation by gap genes and its importance for setting the boundaries of various other stripes; in contrast, odd resolves fairly late and is patterned mainly by cross-regulation. In Bombyx (Nakao 2015), the relative importance of odd and eve seems to be reversed, suggesting some contingency in how the early pair-rule gene network decomposes once gap genes become involved.
line 261 – "has changed" could imply Drosophila represents the ancestral state; neutral language such as "is different" would avoid this.
line 261 – since Eve represses slp in Drosophila, this implies regulatory evolution between the two species. Note also that there are differences across insects in which set of slp stripes appear first. Nasonia resembles Tribolium (Choe and Brown 2007) in this respect rather than Drosophila. Drosophila has numerous partially redundant slp enhancers (Fujioka and Jaynes 2012), so maybe this interspecies variation is not particularly surprising.
line 263 – "pair-rule", not "segmental". More could be made of this finding, since e75a gives a pair-rule phenotype in Oncopeltus when knocked down (Erezyilmaz et al 2009). The expression in Nasonia suggests that its segmentation role may be extend outside Hemiptera, although this would need to be functionally tested.
line 267 – clarify that the lack of cross-regulation is only at early stages (partly because they are not yet expressed).
line 266-270 – both the conserved aspects (primary pair-rule gene phasing) and diverged aspects (secondary pair-rule gene expression, e75a) should be given appropriate weight here.
line 270 – be careful about how "network topology" vs "network behaviour" are used.
line 271 – see comment re line 48.
line 271-282 – note that even if a region of the embryo went through the whole sequence, this would be entirely consistent with gap-driven (and indeed simultaneous) patterning. (Imagine simulating Drosophila patterning but delaying late network activation; the primary stripes would shift by a greater amount and the individual cells would transit through the entire pair-rule sequence.) The key issue is how periodicity is initially generated (from posterior oscillations or de novo), not the subsequent dynamics of the stripes.
line 283-286 – are you able to say anything about how much cell movement occurs in the Nasonia embryo during these stages?
line 293-295 – note that in Drosophila the dynamic expression of the PRGs is due both to the anterior movement of gap gene expression AND the intrinsic dynamics of the network. The data from Nasonia (dynamic PRG stripe resolution and significant anterior shifts, but stripes emerge de novo in a fairly ad hoc manner rather than emanating from a posterior oscillating zone) suggest a similar mechanism, just with a pronounced AP timelag in pattern maturation.
line 299-323 – the search for topologies that could produce an h/eve/runt/odd/h sequence is useful, but I think could be framed differently, as I don't think the motivating assumption of sequential segmentation / autonomous oscillations is particularly sound. Elucidating the various sets of regulatory interactions consistent with the dynamics of the primary pair-rule genes tells us how much the observation of conserved expression phasing/dynamics constrains the topology of the network, and therefore how similar to the Drosophila network the Nasonia network is likely to be. (Ie how much developmental systems drift could feasibly have occurred?)
line 304-305, as with line 255, the wrong sequence is listed here.
line 309-310 – can we draw the lesson from this that we should expect at least one pair-rule gene to lose its cross regulation (replaced by gap regulation) during any transition to long-germ segmentation? ie that we should expect the segmentation clock network to evolve into a hierarchical network (similar to the Drosophila early pair-rule gene network), otherwise pair-rule gene cross regulation would tend to erase the imposition of patterning by the gaps?
figure 4 legend – "stable gene phasing" re K-M is potentially confusing, since these are transient rather than stable patterns. Showing the transcript patterns without the protein patterns is also an issue, since without them it is difficult to understand how the pattern is produced by the network.
line 328 – these are changes to late gene expression rather than changes to the late network.
line 343 – be careful with the parasegment / segment terminology. odd numbered parasegments are roughly equivalent to even numbered segments, and vice versa. I think this should read "even-numbered parasegment".

© 2022. Published by The Company of Biologists under the terms of the Creative Commons Attribution License (https://creativecommons.org/licenses/by/4.0/).

fig 5D – shift the alignment of the eve5 stage to the right by a couple of cells so that it is consistent with the pattern resolution in the later panels. It would be good to include e75a in this schematic too. Can you comment on how the diagram compares with Rosenberg 2014?
line 361-368 – how do you know the picture derived from in situ snapshots is “stable”? It seems that time lags involved in Eve synthesis and decay could explain both of these phenomena; the pair-rule gene dynamics mean that protein and transcript domains will not be identical.
line 377-378 – what is the reasoning for this statement? The way that pair-rule gene patterns mature in other species (eg Stragamia) also supports this idea, even if they have not been explicitly analysed in this context.
line 386-410 – several earlier comments are relevant to the discussion here.
line 416-417 – prd and slp are secondary PRGs.
line 421-422 – the statement in lines 309-310 argues against this. See also the comment re lines 299-323.
line 435-8 – this could be phrased better, it’s incorrect as currently stated.
line 453 – pair-rule gene expression in myriapods and chelicerates suggests this involvement is considerably older!
line 451-464 – it would be useful to have some discussion of how the network topology could be functionally tested; ie what observations would we expect upon knocking down particular PRGs? Which are the crucial interactions to test to assess the degree of conservation with Drosophila? Also, how do these results relate to the a wider comparative context (PRG patterns in Tribolium, Bombyx, Oncopeltus, for example)?
line 468-469 – how might the changed expression of slp and prd be explained?
line 475 – why not older?
line 479 – “endless variety” seems overstated.
line 499 – check sentence.
line 510-512 – more details about image acquisition would be helpful here.
line 522 – check spelling.
reviewer: Erik Clark

First revision

<u>Author response to reviewers' comments</u>

Dear Cassandra,

Thank you for your kind comments on our manuscript. We have extensively reworked the paper at your, and the reviewers' suggestions. I apologise for the length of time it has taken to revise the manuscript, it has been difficult to coordinate writing between the two of us while posted at opposite ends of the planet in a pandemic.
Below we address each of the reviewer's comments in turn, and we also provide a marked up version of the text to help you trace where we have made changes. The revisions suggested by reviewer two has led to an increase in the number of figures, and in the word count of the manuscript.

Thanks again, and best wishes.

Peter

Reviewer 1

Descriptions in figure legends could be a little more complete such they stand alone and don't rely on having read the cited papers.

We have revised the figure legends to include more details.

© 2022. Published by The Company of Biologists under the terms of the Creative Commons Attribution License (https://creativecommons.org/licenses/by/4.0/).

Reviewer 2

For the text, I would recommend that the authors think about how best to walk readers through a topic which can seem dauntingly complex, including providing sufficient background context to understand the findings, and properly emphasising the evolutionary significance of the key results. Some of the less essential results, such as some of the model analysis, could be moved to the supplementary information, to maintain the flow of the text.

We have edited the text extensively to ensure we are communicating our results effectively. We have decided to include the model analysis in the text as we think it is a key outcome of the manuscript

More specifically, it would be helpful if the introduction could be revised to provide more context about: 1) the phylogenetic position of Nasonia and the distribution of different modes of segmentation among arthropods; 2) previous work on Nasonia segmentation; 3) an overview of the relevant components of the Drosophila segmentation network (primary PRGs vs secondary PRGs vs segment-polarity genes, early vs late expression patterns and dynamics, timer genes); 4) how these have been proposed to relate to an ancestral clock and wavefront mechanism;
5) key questions to be investigated.

We have revised the Introduction to expand the introduction of these issues.

For the results, in my opinion a more logical order of presentation would be to first present all the pair-rule gene expression data, including summary schematics, commenting on 1) the time lag across the AP axis, 2) the anterior shifting and spatial compression of the pattern over time, and 3) the overt similarities and differences with pattern resolution in Drosophila. Then 1) present the timer gene data to provide a mechanistic explanation for the time lag, 2) quantify the shifts/compression and explain the patterning dynamics as a consequence of the timer gene expression using simple simulations, and 3) analyse which aspects of PRG regulation are likely to be conserved / divergent with respect to Drosophila (and potentially other insects), noting how these hypotheses could be functionally tested where necessary.

We have reorganised the text as suggested

Throughout, care should be taken to clearly distinguish between 1) the mechanism of periodicity generation, 2) the dynamics of pattern resolution, and 3) time differences in pattern maturation along the AP axis, when discussing modes of segmentation. For the take away message, I think it would be more informative to focus more on the overall pattern of network conservation and divergence (eg regulation by timer genes, primary PRG phasing, parasegment boundary expression all fairly conserved; timer gene axis dynamics and the transition to segmental expression are not) rather than emphasising only the conserved aspects.

We have extensively revised the text along these lines, especially making it clear what is conserved and what is not. Please see the marked-up version of the text for details.

I would also recommend that the authors rework and polish the figures, again with the aim of walking the reader through the key results. Overcrowded figures should be split up, layout/colours/labels/annotations optimised for information communication, embryo orientation made more consistent, enlarged image crops used to more clearly communicate stripe phasing etc. I was also surprised that there was not much use made of the multiplexed nature of the image data in the main display figures; this seems a missed opportunity. To improve SNR, the authors might try experimenting with average projection instead of maximum projection, and/or project over a smaller Z range.

We have extensively revised the figures along the line so the feedback provided. Major changes include

- A different staging system using Roman Numerals rather than staging by eve stripe number to improve clarity

© 2022. Published by The Company of Biologists under the terms of the Creative Commons Attribution License (https://creativecommons.org/licenses/by/4.0/).

- We have added one new figure as the second figure of the paper, which helps with the flow of the Results and helps reduce the complexity of Figure 1
- We did not use multiplexed images extensively because we felt it made the figures more complicated, rather than less. Eg. the embryos in Fig 2 are visually much simpler in greyscale, and the spatial patterns can be judged reasonably well by eye. Fig 1 is sufficiently complicated as-is. Figs 3-4 do use multiplexed images.
- We have experimented with average rather than maximum projection and found the results worse for our embryos

The discussion should also be revised in light of the changes made to the rest of the manuscript.

The discussion has been revised as suggested.

title / abstract –these should be revised to more accurately reflect the content/significance of the paper.
We have modified the title to reflect our astonishment at how similar *Nasonia* and *Drosophila* seem to be, at a deep level - at the rules/dynamics of patterning. Title is now 'The *Nasonia* pair rule gene regulatory network retains its function over 300 million years of evolution'.

line 14 – why these three species in particular?
We have reworded to "This idea needs to be tested in a wider variety of species, and the nature of the gene regulatory network (GRN) underlying this model has not been tested."

line 29 – I would argue "extensively" applies only to Drosophila.
We have removed this adjective.

line 31 – should Akam 1989 be Akam 1987 (the Development review) instead?
We have changed this citation.

line 33 – there are others as well, such as maternal Hb and the terminal system.
We have noted other maternal protein play a role

line 42 – that this is a spectrum rather than a dichotomy has been stressed elsewhere, including in the reviews just cited.
We have reworded this to cite Peel and Akam 2003 and Clark et al. 2019 as pointing out this is a spectrum.

lines 46-48 – I would suggest a slightly different definition, i.e. that stripes appear de novo (arguing for periodicity being generated by instructive positional cues rather than segmentation clock), while the tissue / segment pattern matures in an anterior to posterior progression. The periodicity generation and temporal regulation aspects of patterning are therefore contrasted and kept distinct.

We have modified the text to read "That is, stripes of segmentation gene expression appear de novo, one after the other, but are not produced from the posterior of the embryo."

line 48 – "Nasonia" is used throughout as a plural. Is this standard?
Yes

line 48 – It's worth being more circumspect here; I don't think that "sequential" (ie clock-driven) segmentation in the posterior is solidly established. Based on PRG expression dynamics and various knockdown phenotypes, Rosenberg et al 2014 proposed that Nasonia possesses a "dual" mode of segmentation, which is "Drosophila-like" in the anterior (pair-rule stripes patterned by gap genes) and "Tribolium-like" in the posterior (pair-rule stripes patterned by dynamic cross-regulation, by the remnants of segmentation clock). However, although Nasonia stripes do appear (approximately) sequentially, they generally form de novo, arguing against a segmentation clock mechanism. More recently, I have proposed that, even in Drosophila, pair-rule stripe phasing is dynamically established by the remnants of a segmentation clock, meaning that instructive gap gene cues and a large role for pair-rule gene cross- regulation are not mutually exclusive. I think there are at least three separable issues here: 1) how is periodicity established (by gap genes or

© 2022. Published by The Company of Biologists under the terms of the Creative Commons Attribution License (https://creativecommons.org/licenses/by/4.0/).

by a segmentation clock?); 2) what is the rate/time delay of pattern maturation along the tissue (ie how are the timer genes expressed); 3) how important are pair-rule gene expression dynamics in establishing stripe boundaries / phasing (on a spectrum from entirely PRG-regulated to entirely gap gene regulated).

We think the evidence is in favour of clock-like patterning of the *Nasonia* posterior - especially the final eve stripe (not the ones emerging from eve stripe 6 onwards). This is mostly because of the kinematic waves of *hairy* and *odd* in the posterior (most convincing in Rosenberg et al's in situs). But obviously this hasn't been proven! In particular this evidence is consistent with a damped oscillator, or an oscillator patterned by gap genes. In our opinion, whether the *Nasonia* posterior is a true oscillator like *Tribolium*, or a damped/gap driven oscillator like *Drosophila*, isn't really relevant for this paper, and questions about the difference between the two are best approached in eg. *Drosophila* where better tools are available. Either way the predictions for eg. timer gene expression would be the same.
I'd also like to note that segmentation in the stripe 6 region appears to be very different to the rest of the embryo- simultaneous, for one thing! (We don't go into this in depth in the main text, because 1) it complicates an already complicated story, 2) we don't understand it well, and 3) it isn't necessary for the argument we are trying to make). This is different to what is happening in the very posterior.
In the anterior, there is no evidence for patterning via sustained oscillations of a segmentation clock. Our data doesn't speak to whether the oscillatory behaviour of the early network is gap driven vs pair rule gene driven- we're more interested in the dynamics of the process itself and how these vary (or don't vary!) over evolutionary time.

To resolve points 1-3 above, we have added the following text at the end of this paragraph.
'The initial pattern could come from periodic pair rule gene expression (a segmentation clock), and/or gap gene expression.'

We have not addressed the reviewers point 3, because we do not have the functional data needed to do sot.

We prefer the simpler definition we have retained, in part because of positive feedback suggesting the simpler definition makes the idea easier to understand.

line 53 – the meaning of 'the same molecular process' is unclear.
We have changed this to GRN

line 54 – it is potentially confusing that here the text talks about an individual instance of segmentation being controlled by two different networks, and earlier about different modes of segmentation being controlled by a single network.
We have changed this to 'GRNs' to improve clarity.

line 65 – Sox21b (not D) is involved in Parasteatoda segmentation.
We have changed this to read 'D (or Sox21b in spiders) is required for normal expression…'

line 77 – "switch" language doesn't seem appropriate, since it is previously argued that sequential vs simultaneous timing is a spectrum.
We have changed this sentence to read "expression patterns of timer genes controls the difference between sequential and simultaneous segmentation"

line 79-90 – this section is subsuming a variety of conceptually different things under the heading of "multifunctional GRN", which I think it would be clearer to distinguish between. First, a single network motif (eg AC/DC) which can give rise to qualitatively different behaviours depending on the quantitative strength of particular component interactions. Second, context-specific gene networks where the regulation of individual genes is strongly dependent on tissue / stage-specific TFs (eg different timer genes activating different pair-rule gene enhancers). Third, a gene network that is deployed in different ways across different tissues / organisms, although its logic and intracellular dynamics are conserved (the pair-rule gene network in different insect species, potentially).

© 2022. Published by The Company of Biologists under the terms of the Creative Commons Attribution License (https://creativecommons.org/licenses/by/4.0/).

We have updated this section to expand on different uses of the term “function” in biology, to make our meaning clearer.

line 114 – how was the gene set (including e75a) decided? Which genes have been looked at previously in Nasonia and which are unique to this paper?
We have added the following text -‘We stained for the known Drosophila pair rule genes, plus Nv-e75A which is involved in Oncopeltus segmentation. All of these except for Nv-e75A and Nv-slp have been previously described; comprehensive descriptions are available for Nv-eve, Nv-odd, Nv-hairy, Nv-runt, Nv-wg, and Nv-prd (Rosenberg2014, Keller2010, Olesnicky2007a). We build upon this work by elaborating the timing of these events, by reporting the expression of Nv-slp and Nv-ftz, and by reporting the relative expressions of genes within the embryo.’

line 115 – specify ectoderm / mesoderm boundary?
We have done this

line 116 – this staging scheme is very confusing, because “eve4” might mean eve stripe 4, or an embryo with 4 eve stripes, none of which is eve4! It would be more intuitive for the reader if eve2 through wg13 were converted to an unified numerical scheme, either in term of time AEL, or just phases/stages 1, 2, …, N.
We have changed the staging scheme to one based on roman numerals throughout the paper.

line 118-163 – it would help the reader if this information was presented one topic at a time. What is the temporal pattern of gene expression in a given region and is this the same across the axis and the same as in other species? How does the timing of gene expression vary along the axis? How does this timing relate to other embryonic events? What are the phasing relationships between the different genes and how do these change over time? Which bits of this information are new in this manuscript vs previously described?
Section 2.1 has been restructured with this in mind, addressing each of these points in order. NB the final point- about what information is new- is addressed above.

line 145 – see comment about line 48.
Throughout the text, we’ve replaced “behaviour” with activity-function, which is defined in the Introduction. We hope this makes our meaning clearer

line 153-156 – this definition is not straightforward to apply when gastrulation occurs progressively along the AP axis rather than simultaneously. (Note also that the 15th stripe of eve expression forms after gastrulation in Drosophila.)

Based on previous publications definitions we believe our text is both clear and correct.

line 157 – since the PRG stripes resolve over time, it is preferable to quantitate the width of the pair-rule repeat rather than the width of the stripes themselves. (Eg peak to peak or trough to trough.) In Fig 1B, is there a more reliable posterior anchor that can be used instead of eve expression? The posterior border of the final eve stripe shifts quite considerably in Drosophila, for example.
We have also indicated the cells from peak to peak/front of stripe to front of stripe in Fig 2B. We have decided to remove the kymograph from the figure, for the following reasons.

1) Stripe compression and stripe movement is now all addressed in the same figure and up-front, so there is no need for an additional panel communicating the same things.
2) We are dissatisfied with the alignment of the kymograph- Reviewer 2 is correct to point out that this posterior anchor is not very good (indeed, the sixth eve stripe moves forward in Nasonia). We thought about using other markers. Ie the posterior of the embryo- but this would magnify any inconsistencies in how we are measuring along the curvature of the embryo. Or the posterior wg stripe, but we have no guarantee this doesn’t also move.
3) Without accurate temporal information (timed lays), the ordering and alignment of the kymograph is based on our best guess, and cross-correlation to other gene expression patterns, not necessarily the time AEL. (We would be unable to do precisely timed lays, because Nasonia take ~20 minutes to lay a clutch of eggs anyway).

© 2022. Published by The Company of Biologists under the terms of the Creative Commons Attribution License (https://creativecommons.org/licenses/by/4.0/).

line 165 – it might be more straightforward to talk about the compression of the pattern over time rather than the "expanded initial pattern".
We have changed this wording to "compression of the pair rule pattern over time" here

line 168 – which model?
Reworded to be more clear

line 169-172 – the previous description of Nv-cad by Olesnicky et al should be cited.
Done

line 179 – this unusual eve expression should be flagged earlier, when the PRG patterns are described. Channel merges should be included in fig 2 to show the spatial registration of the different patterns.
We have re-phrased a portion of Section 2.1 to discuss segmentation in this stripe more explicitly.

line 181-189 – this is an important insight, which is complicated somewhat by being presented in the context of the full Drosophila pair-rule model. I would lean towards presenting simpler clock-and-wavefront simulations to explain the point, ie something that looks more like figure 6.
We agree, but did not have the time to perform more simulations that would not have changed the results of the paper.

line 190-209 – nice functional data, which should be mentioned in the abstract. It is interesting that the opa knockdown causes an asegmental phenotype, whereas in Drosophila the ftz-dependent boundaries are lost but the prd-dependent boundaries still form. A change in the relative importance of prd and opa for activating segment- polarity gene expression would be consistent with the delayed prd expression seen in Nasonia vs Drosophila.

We have included this information in the abstract along with other edits, please see the marked up version.

line 216 – "ventral ends of the embryo" unclear.
Removed this as it is superfluous

line 217-221 – similar spatiotemporal dynamics are seen in the equivalent (tail / eve 15) region of the Drosophila embryo – see eg the supplementary information in Clark and Peel 2018. Think about the colour choices in Fig 3; green/yellow, green/cyan, and red/magenta are all hard to discriminate between.
We have changed phrasing to point this out.
We have changed the color schemes in Fig 3 to be easier to follow, and added more visual aids to this figure.

line 222 – can more be said about what is going on in the stripe 6 region? Exceptions are interesting.
We have added the following text (after the description of anterior timer gene patterns)

'The middle region of the Nasonia embryo, stripe 6, does not follow the above sequence of timer genes, either spatially or temporally. In this region of the embryo, Nv-cad and Nv-D RNA are never expressed; only Nv-opa RNA. How this stripe achieves stripe splitting without cad and D is unclear.'

line 226-227 – note that the early Drosophila PRG network involves oscillatory dynamics, but does not involve an intact oscillator per se. It is worth addressing the question of whether Nasonia retains an intact oscillator/segmentation clock in the posterior as has been suggested (I don't see compelling evidence for this) vs exhibiting oscillatory dynamics which are presumably evolutionary remnants of such an oscillator (there seems to be good evidence for this across most of the axis).

We have added the following text:
Whether the Nasonia posterior is patterned by an oscillatory GRN is unclear, and our data does not address this. The kinematic waves of odd in the posterior argue for an oscillator (Rosenberg2014)

© 2022. Published by The Company of Biologists under the terms of the Creative Commons Attribution License (https://creativecommons.org/licenses/by/4.0/).

line 230 – I think this section is more complicated than it needs to be. In Drosophila, Opa acts as a pioneer factor at numerous PR and SP gene enhancers (Soluri et al 2020), thereby precipitating about stable, segmental PR and SP gene expression, in part by changing the regulatory interactions between the PRGs (Clark and Akam 2016, Clark 2017). The spatiotemporal dynamics and changing expression relationships (eg eve/slp) of the Nasonia PRGs, combined with the spatiotemporal dynamics of Nv-opa, are consistent with a similar role for Opa being conserved in Nasonia segmentation.
We have opted to leave this section as-is, so our argument does not rely too heavily on comparison to *Drosophila*. We think it better to discuss our data, interpret that data to mean there are two GRNs acting in *Nasonia* segmentation (using similar criteria to Clark and Akam 2016), which looks like Drosophila. It is tempting to jump straight to the comparison, but we believe we have to interpret what we see, especially when we are making the argument that this is like Drosophila, rather than jumping straight to interpreting this as 'like Drosophila'. We appreciate this is a fine point, but we think it is a less biased interpretation of the data - stating our interpretation independently of what *Drosophila* is doing - is important so as not to interpret everything as being like *Drosophila*.

line 246-247 – "topology" or "structure" rather than behaviour?
We have changed this to read activity-function (defined in the introduction)

line 248-249 – see the comment re line 54. It's probably useful to explicitly address the multiple tissue-specific networks vs single network with subnetworks terminology/usage issue somewhere to avoid confusion.
This is problematic as we are inferring networks. We have tried to address the issue by being more careful about what we mean by 'function' in these networks drawing on the idea of activity-function.

We have re-organised the early network section to address a number of points which relates to reviewers comments below

1) First address the conservation in gene phasing between Nasonia and Drosophila, and highlight differences
2) Mention forward stripe shifts
3) Discuss pattern maturation over time (as we'd expected based on timer gene expression)
4) Say this is the same in the posterior
5) Mention relative timings of gene expression (and implications for network structure)
6) Discuss the implications of these findings for the topological structure of the network - does it require changes to Drosophila network ?
7) Discuss the screen for networks in terms of how this pattern + possible requirement for clocklike segmentation constrains GRN topologies

We think this addresses many of the comments below

line 255 – runt should be before odd+ftz.

Yes, we made an error here- this is now corrected

line 255-8 – why colour gradients rather than line plots?
We found line plots so complex as to be uninterpretable especially without smoothing

line 258-260 – can anything be inferred from the order in which the stripes of different genes resolve? In Drosophila, the eve pattern resolves before the other pair-rule genes, consistent with eve's extensive regulation by gap genes and its importance for setting the boundaries of various other stripes; in contrast, odd resolves fairly late and is patterned mainly by cross-regulation. In Bombyx (Nakao 2015), the relative importance of odd and eve seems to be reversed, suggesting some contingency in how the early pair-rule gene network decomposes once gap genes become involved.

We have added the following text - 'The relative timing of gene expression is similar in both these regions of the embryo. Throughout the axis, Nv-hairy is the first gene to be segmentally expressed, followed by Nv-odd, Nv-runt, and Nv-eve. This implies that 1) a similar patterning process is acting

© 2022. Published by The Company of Biologists under the terms of the Creative Commons Attribution License (https://creativecommons.org/licenses/by/4.0/).

in different regions of the embryo, just with different dynamics, and 2) that Nv-hairy and Nv-odd play an important role in establishing the initial pair rule pattern. This could be further tested by eRNAi followed by analysis of gene expression patterns.'

line 261 – "has changed" could imply Drosophila represents the ancestral state; neutral language such as "is different" would avoid this.
Changed this to read "is different"

line 261 – since Eve represses slp in Drosophila, this implies regulatory evolution between the two species. Note also that there are differences across insects in which set of slp stripes appear first. Nasonia resembles Tribolium (Choe and Brown 2007) in this respect rather than Drosophila. Drosophila has numerous partially redundant slp enhancers (Fujioka and Jaynes 2012), so maybe this interspecies variation is not particularly surprising.
We have made the differences in Nasonia with respect to secondary pair rule expression more clear. We have added the following text to the Discussion:
Moreover, the co-expression of Nv-slp and Nv-eve implies regulatory evolution. In this way, Nasonia resemble Tribolium, not Drosophila, where the even-numbered stripe develops first.

line 263 – "pair-rule", not "segmental". More could be made of this finding, since e75a gives a pair-rule phenotype in Oncopeltus when knocked down (Erezyilmaz et al 2009). The expression in Nasonia suggests that its segmentation role may be extend outside Hemiptera, although this would need to be functionally tested.
Changed pair rule to segmental.

The pair rule expression of Nv-e75A implies that this gene may be required for segmentation in Nasonia, the first evidence of its use in pair rule patterning outside of bugs Erezyilmaz2009, Reding2019, Hernandez2020.

line 267 – clarify that the lack of cross-regulation is only at early stages (partly because they are not yet expressed).
We have made this change.

line 266-270 – both the conserved aspects (primary pair-rule gene phasing) and diverged aspects (secondary pair- rule gene expression, e75a) should be given appropriate weight here.
We have added the phrase 'We also observed differences in gene expression compared to Drosophila, …'

line 270 – be careful about how "network topology" vs "network behaviour" are used.
We have changed this to read activity-function (defined in the introduction)

line 271 – see comment re line 48.
We've modified this in response to the comment on line 48

line 271-282 – note that even if a region of the embryo went through the whole sequence, this would be entirely consistent with gap-driven (and indeed simultaneous) patterning. (Imagine simulating Drosophila patterning but delaying late network activation; the primary stripes would shift by a greater amount and the individual cells would transit through the entire pair-rule sequence.) The key issue is how periodicity is initially generated (from posterior oscillations or de novo), not the subsequent dynamics of the stripes.
We have removed this section

line 283-286 – are you able to say anything about how much cell movement occurs in the Nasonia embryo during these stages?
We did live imaging to address this, but due to time constraints never got the sample size needed. Perhaps the next paper?

line 293-295 – note that in Drosophila the dynamic expression of the PRGs is due both to the anterior movement of gap gene expression AND the intrinsic dynamics of the network. The data from Nasonia (dynamic PRG stripe resolution and significant anterior shifts, but stripes emerge de novo in a fairly ad hoc manner rather than emanating from a posterior oscillating zone) suggest a

© 2022. Published by The Company of Biologists under the terms of the Creative Commons Attribution License (https://creativecommons.org/licenses/by/4.0/).

similar mechanism, just with a pronounced AP timelag in pattern maturation.
We have addressed this point in our revision of the early GRN network.

line 299-323 – the search for topologies that could produce an h/eve/runt/odd/h sequence is useful, but I think could be framed differently, as I don't think the motivating assumption of sequential segmentation / autonomous oscillations is particularly sound. Elucidating the various sets of regulatory interactions consistent with the dynamics of the primary pair-rule genes tells us how much the observation of conserved expression phasing/dynamics constrains the topology of the network, and therefore how similar to the Drosophila network the Nasonia network is likely to be. (Ie how much developmental systems drift could feasibly have occurred?)
We have reframed these experiments as suggested

line 304-305, as with line 255, the wrong sequence is listed here.
Fixed.

line 309-310 – can we draw the lesson from this that we should expect at least one pair-rule gene to lose its cross regulation (replaced by gap regulation) during any transition to long-germ segmentation? ie that we should expect the segmentation clock network to evolve into a hierarchical network (similar to the Drosophila early pair-rule gene network), otherwise pair-rule gene cross regulation would tend to erase the imposition of patterning by the gaps?
We don't think so- we excluded any network containing the core oscillatory network, so presumably eg. gap inputs to hairy could be redundant with hairy regulation by runt etc.

figure 4 legend – "stable gene phasing" re K-M is potentially confusing, since these are transient rather than stable patterns. Showing the transcript patterns without the protein patterns is also an issue, since without them it is difficult to understand how the pattern is produced by the network.
We have removed "stable" from the phrase "stable gene phrasing".
We have not included protein patterns, as we do not think this makes it easier to understand.

line 328 – these are changes to late gene expression rather than changes to the late network.
Fixed.

line 343 – be careful with the parasegment / segment terminology. odd numbered parasegments are roughly equivalent to even numbered segments, and vice versa. I think this should read "even-numbered parasegment". Thank you yes, this has been changed.

fig 5D – shift the alignment of the eve5 stage to the right by a couple of cells so that it is consistent with the pattern resolution in the later panels. It would be good to include e75a in this schematic too. Can you comment on how the diagram compares with Rosenberg 2014?

We agree that shifting the alignment of the eve5/stage iii embryo would be more parsimonious with the following panel. However this would imply that a shifted pair rule pattern gives rise to an identical segment polarity pattern, something we feel is highly unlikely. We know that the registration of the following stages (iv onwards) is correct with regards to the segment polarity pattern because slp is a very strong and persistent marker. To investigate this further, we went through the data again and accordingly have revised the positioning of runt and ftz, and therefore re-ran simulations from stage iv/eve8. This does not change the results of the paper.

E75a has been added to the figure.

We have added the following text: Note that our positioning of the runt stripes disagrees with Rosenberg2014: we place the strong runt stripe in the even-numbered parasegment (Fig 7D,E).

line 361-368 – how do you know the picture derived from in situ snapshots is "stable"? It seems that time lags involved in Eve synthesis and decay could explain both of these phenomena; the pair-rule gene dynamics mean that protein and transcript domains will not be identical.
We make the assumption that protein and RNA degradation takes about the same amount of time for each gene, a simplifying assumption also employed in Clark 2017. Obviously, this assumption may not hold, in which case our argument is not valid. We have made this assumption explicit in-

© 2022. Published by The Company of Biologists under the terms of the Creative Commons Attribution License (https://creativecommons.org/licenses/by/4.0/).

text as below

Accordingly, we have added the following text:
'This analysis assumes similar production and degradation rates for all genes: if this does not hold, then these predictions will not either.'

line 377-378 – what is the reasoning for this statement? The way that pair-rule gene patterns mature in other species (eg Strigamia) also supports this idea, even if they have not been explicitly analysed in this context.
Very true- we have removed the second half of this sentence.

line 386-410 – several earlier comments are relevant to the discussion here.
We have removed the repetition of the timer gene hypothesis here which this comment refers to in the interest of length

line 416-417 – prd and slp are secondary PRGs.
This has been changed as suggested.

line 421-422 – the statement in lines 309-310 argues against this. See also the comment re lines 299-323.
We have revised both lines so they are consistent

line 435-8 – this could be phrased better, it's incorrect as currently stated.
We have changed this to 'The output of the late network, expression of the segment polarity genes, is still largely identical to \textit{Drosophila}, consistent with previous empirical and modelling work showing the segment polarity network is stable and well conserved (VonDassow2004, Choe2009, Janssen2013, Green2013, Vellutini2016, Auman2018, Clark2019). We interpret this as meaning the activity-function of the late network, in this case the output/attractors of the system, is unchanged. '

line 453 – pair-rule gene expression in myriapods and chelicerates suggests this involvement is considerably older!
We have added "and may be considerably older"

line 451-464 – it would be useful to have some discussion of how the network topology could be functionally tested; ie what observations would we expect upon knocking down particular PRGs? Which are the crucial interactions to test to assess the degree of conservation with Drosophila? Also, how do these results relate to the a wider comparative context (PRG patterns in Tribolium, Bombyx, Oncopeltus, for example)?

We have provided predicted observations of knock-down of PRG in Nasonia, and pointed out the crucial ones to test

We have not added speculation about the wider comparative context as much of the work required hasn't been done outside Drosophila. We don't feel we can speculate more broadly than this system appears to be conserved between Drosophila and Nasonia, implying it may be conserved in most holometabolous insects.

line 468-469 – how might the changed expression of slp and prd be explained?
We have carried out no experiments to investigate this.

line 475 – why not older?
We have changed this to be "deep in the arthropod lineage"

line 479 – "endless variety" seems overstated.
We have changed this to 'wide variety'

line 499 – check sentence.
We have fixed this sentence

© 2022. Published by The Company of Biologists under the terms of the Creative Commons Attribution License (https://creativecommons.org/licenses/by/4.0/).

line 510-512 – more details about image acquisition would be helpful here.
We have added more details

line 522 – check spelling.
repaired

Second decision letter

MS ID#: DEVELOP/2021/199632

MS TITLE: The Nasonia pair rule gene regulatory network retains its function over 300 million years of evolution

AUTHORS: Shannon Taylor and Peter Dearden

I have now received a referee reports on the above manuscript, from one of the reviewers of the original MS. I was waiting for several months for a second report from one of the original reviewers, but they have been unresponsive to requests to follow through with their report, which is why there has been this long delay. I therefore read the MS carefully myself a second time, and, together with the report we do have in hand, have reached a decision. The referees' comments are appended below, or you can access them online: please go to BenchPress and click on the 'Manuscripts with Decisions' queue in the Author Area.

The overall evaluation is positive and we would like to publish a revised manuscript in Development, provided that the referees' comments can be satisfactorily addressed. Please attend to all of the reviewers' comments in your revised manuscript and detail them in your point-by-point response. If you do not agree with any of their criticisms or suggestions explain clearly why this is so. In addition to the points of the reviewer below, please also make the minor correction of 'Eric Clark'¤ to 'Erik Clark'¤ in your acknowledgements section.

We are aware that you may currently be unable to access the lab to undertake experimental revisions. If it would be helpful, we encourage you to contact us to discuss your revision in greater detail. Please send us a point-by-point response indicating where you are able to address concerns raised (either experimentally or by changes to the text) and where you will not be able to do so within the normal timeframe of a revision. We will then provide further guidance. Please also note that we are happy to extend revision timeframes as necessary.

Reviewer 2

Advance summary and potential significance to field

The authors engaged satisfactorily with my extensive comments from the first round of review, and carried out a comprehensive reorganisation and revision of the text. The manuscript is much improved and the authors' conclusions about the Nasonia segmentation network are, in my judgement, sound.

I have some minor notes regarding wording that could be made clearer, edits to figure legends etc. These should be trivial for the authors to address as they see fit.

- Erik Clark

Comments for the author

[NB: line numbers refer to the tracked changes version of the manuscript.]

© 2022. Published by The Company of Biologists under the terms of the Creative Commons Attribution License (https://creativecommons.org/licenses/by/4.0/).

l210 - "the end of the embryo, 85-90% egg length" - 'a region towards the posterior of the embryo, at 85-90% EL' might be clearer, since the very end of the embryo is only described in the next paragraph.
l215 - "Shortly after this, the 11th Nv-eve RNA stripe emerges from the anterior of the 6th eve RNA stripe" - this was confusing to me - do stripe numbers refer to position in the embryo or order of appearance? Is there different numbering for pair-rule and segmental phases of expression?
l263 - "As this, as in other regions of the embryo..." - check sentence.
l301 - it could be helpful to distinguish this terminal wg domain from the segmental wg stripes.
l309 - note this is from posterior to anterior.
l317 - "assumes that there are two different pair rule GRNs in Nasonia" - it could be helpful to the reader to explain this a little more fully - ie that the overall Nasonia GRN contains two different pair-rule GRNs that are contextually activated by timer genes and show qualitatitively different activity functions.
l345 - "We then investigated the conservation of the two pair rule networks" - specify relative to Drosophila.
l442 - "Unlike the hypothetical network, this potential network" - it would be clear to spell out explicitly which two networks are being referred to here.
l464 - "Changes to the Nasonia late gene expression are as follows" - specify relative to Drosophila.
Section 2.5, re the comment "We make the assumption that protein and RNA degradation takes about the same amount of time for each gene, a simplifying assumption also employed in Clark 2017. Obviously, this assumption may not hold, in which case our argument is not valid."
This is true. But, to clarify, my point to which this was a response was actually a different one. If there were offsets between protein and transcript at the timepoint at which the simulations were started, it is possible that this could provide the missing spatial info for pairs of cells with identical transcript expression, without the necessity to invoke an additional gene. I don't think this would affect the authors' conclusions about odd and runt regulation, though.
l517 - "The general organization of the pair rule genes into two networks is likely conserved in Nasonia" - probably clearer to refer to the interactions between the genes rather than the genes themselves, as these are shared between both networks.
l518 - "though there are changes to the genes expressed in the early network" - unclear as written whether this refers to the identities of the genes or their manner of expression.
l543 - "Broad pair rule stripes (an expanded spatial pattern) correct for the temporal pattern" - as a side note, I would tend to interpret this the other way around, as the temporal pattern being necessitated by the broad early stripes! However, if we are just talking about the mechanism of pattern formation (and not selection pressures/constraints on pattern formation) these alternatives are logically equivalent.
l561 - "The process could be the same or different in these regards." Unclear as written whether 'process' refers to the biological process under study or the process of assessing GRN similarity.
l563 - "We think that the Nasonia network is the same with regards to..." - specify with respect to Drosophila.
l613 "(dynamic behaviour of the primary pair rule genes; and segment polarity genes)" - are there words missing before segment polarity genes?
l690 "small detection wavelengths" - narrow emission collection windows, maybe?

fig 1 legend states "All embryos are... oriented anterior left and dorsal up". I am not experienced at looking at Nasonia, but it looks to me as though some of the embryos are lateral views, some dorsal views, and some ventral (also for fig S3)? The meaning of the red annotations (sim expression) is not described. The orientations of the embryos could be checked and the legend updated to address these points. Figure annotations showing the identity (number) of key stripes would also be helpful.
fig 2 legend states "Nasonia segment polarity genes are expressed in a one-cell repeat" - the meaning of this was unclear to me - one-cell-wide stripes within a four-cell repeat?
fig 6 legend states "Repressive interactions are read X to Y, thas is, run represses eve" - currently the repressive arrow connect runt and runt; adjust so that it points to eve.

© 2022. Published by The Company of Biologists under the terms of the Creative Commons Attribution License (https://creativecommons.org/licenses/by/4.0/).

Second revision

Author response to reviewers' comments

Dear Editor,

Thank you for the opportunity to revise our manuscript. We have taken the reviewers suggestions and responded as listed below. We have provided a marked up version of the manuscript as well so you can see where we have changed things.

Best wishes

Peter

Reviewers comments (with our response in red)

l210 - "the end of the embryo, 85-90% egg length" - 'a region towards the posterior of the embryo, at 85-90% EL' might be clearer, since the very end of the embryo is only described in the next paragraph.

We have changed this phrasing as suggested.

l215 - "Shortly after this, the 11th Nv-eve RNA stripe emerges from the anterior of the 6th eve RNA stripe" - this was confusing to me - do stripe numbers refer to position in the embryo or order of appearance? Is there different numbering for pair-rule and segmental phases of expression?

The numbering is taken from Rosenberg 2014 and is by order of appearance. We have changed phrasing to 'eve RNA stripe 11 to make our meaning clearer. We would like to keep the existing naming scheme as it utilizes a published scheme rather than creating a new one.

l263 - "As this, as in other regions of the embryo..." - check sentence.#

We have changed phrasing to 'Here, as in other regions of the embryo…'

l301 - it could be helpful to distinguish this terminal wg domain from the segmental wg stripes.

We have changed phasing from 'overlapping with wg mRNA ' to 'overlapping with the terminal wg domain'

l309 - note this is from posterior to anterior.

Fixed as suggested.

l317 - ". These " - it could be helpful to the reader to explain this a little more fully - ie that the overall Nasonia GRN contains two different pair-rule GRNs that are contextually activated by timer genes and show qualitatitively different activity functions.

We have changed this to read: 'Firstly, it assumes that the Nasonia genome contains two different pair rule GRNs. These GRNs are activated by different sets of timer genes and have qualitatively different activity-functions--- oscillatory vs non-oscillatory expression of genes.'

l345 - "We then investigated the conservation of the two pair rule networks" - specify relative to Drosophila.

We have changed phrasing to read " We then investigated the conservation of the two pair rule networks we have identified, comparing these to Drosophila"

l442 - "Unlike the hypothetical network, this potential network" - it would be clear to spell out explicitly which two networks are being referred to here.

© 2022. Published by The Company of Biologists under the terms of the Creative Commons Attribution License (https://creativecommons.org/licenses/by/4.0/).

Changed phrasing to “Unlike the hypothetical network of Clark2017, our potential network…

l464 - "Changes to the Nasonia late gene expression are as follows" - specify relative to Drosophila.

Changed sentence to read “Changes to the Nasonia late gene expression, relative to Drosophila are as follows.”

Section 2.5, re the comment "We make the assumption that protein and RNA degradation takes about the same amount of time for each gene, a simplifying assumption also employed in Clark 2017. Obviously, this assumption may not hold, in which case our argument is not valid."

This is true. But, to clarify, my point to which this was a response was actually a different one. If there were offsets between protein and transcript at the timepoint at which the simulations were started, it is possible that this could provide the missing spatial info for pairs of cells with identical transcript expression, without the necessity to invoke an additional gene. I don't think this would affect the authors' conclusions about odd and runt regulation, though.

This is an interesting idea but we have not had time or materials (antibodies) to investigate it. Perhaps the next study?

l517 - "The general organization of the pair rule genes into two networks is likely conserved in Nasonia" - probably clearer to refer to the interactions between the genes rather than the genes themselves, as these are shared between both networks.

We have changed this to ‘The general organization of the interactions between pair rule genes into two networks is likely conserved in Nasonia

l518 - "though there are changes to the genes expressed in the early network" - unclear as written whether this refers to the identities of the genes or their manner of expression.

We have changed this to “In addition, though there are changes to patterns of gene expression in the **late** network,” (late network was also a typo!!)

l543 - "Broad pair rule stripes (an expanded spatial pattern) correct for the temporal pattern" - as a side note, I would tend to interpret this the other way around, as the temporal pattern being necessitated by the broad early stripes!
However, if we are just talking about the mechanism of pattern formation (and not selection pressures/constraints on pattern formation) these alternatives are logically equivalent.

Agreed it is impossible to detangle these ideas

l561 - "The process could be the same or different in these regards." Unclear as written whether 'process' refers to the biological process under study or the process of assessing GRN similarity.

Changed to ’gene regulatory process’

l563 - "We think that the Nasonia network is the same with regards to..." - specify with respect to Drosophila.

Corrected as suggested

l613 "(dynamic behaviour of the primary pair rule genes; and segment polarity genes)" - are there words missing before segment polarity genes?

Yes- we have changed this to “dynamic behaviour of the primary pair rule genes; and relative expression of the segment polarity genes”

l690 "small detection wavelengths" - narrow emission collection windows, maybe?

© 2022. Published by The Company of Biologists under the terms of the Creative Commons Attribution License (https://creativecommons.org/licenses/by/4.0/).

Yes- that's the word! Thank you!

fig 1
legend states "All embryos are... oriented anterior left and dorsal up". I am not experienced at looking at Nasonia, but it looks to me as though some of the embryos are lateral views, some dorsal views, and some ventral (also for fig S3)? The meaning of the red annotations (sim expression) is not described. The orientations of the embryos could be checked and the legend updated to address these points. Figure annotations showing the identity (number) of key stripes would also be helpful.

Changed Fig 1 to address these points- adding annotations to indicate dorsally/ventrally oriented embryos, and an inset with stripe names.

fig 2
legend states "Nasonia segment polarity genes are expressed in a one-cell repeat" - the meaning of this was unclear to me - one-cell-wide stripes within a four-cell repeat?

This has been modified for clarity

fig 6
legend states "Repressive interactions are read X to Y, thas is, run represses eve" - currently the repressive arrow connect runt and runt; adjust so that it points to eve.

Modified as suggested.

Third decision letter

MS ID#: DEVELOP/2021/199632

MS TITLE: The Nasonia pair rule gene regulatory network retains its function over 300 million years of evolution

AUTHORS: Shannon Taylor and Peter Dearden
ARTICLE TYPE: Research Article

I am happy to tell you that your manuscript has been accepted for publication in Development, pending our standard ethics checks.

© 2022. Published by The Company of Biologists under the terms of the Creative Commons Attribution License (https://creativecommons.org/licenses/by/4.0/).
